# Unraveling the Specific Regulation of the Central Pathway for Anaerobic Degradation of 3-Methylbenzoate*

**DOI:** 10.1074/jbc.M115.637074

**Published:** 2015-03-20

**Authors:** Javier F. Juárez, Huixiang Liu, María T. Zamarro, Stephen McMahon, Huanting Liu, James H. Naismith, Christian Eberlein, Matthias Boll, Manuel Carmona, Eduardo Díaz

**Affiliations:** From the ‡ Department of Environmental Biology, Centro de Investigaciones Biológicas-Consejo Superior de Investigaciones Científicas, Ramiro de Maeztu 9, 28040 Madrid, Spain,; the § Biomedical Sciences Research Complex, University of St. Andrews, North Haugh, St. Andrews KY16 9ST, Scotland, United Kingdom, and; the ¶ Institute for Biology II, University of Freiburg, 79104 Freiburg, Germany

**Keywords:** Bacterial Transcription, Gene Expression, Microbiology, Protein-DNA Interaction, Transcription Regulation, 3-Methylbenzoate, Azoarcus, Anaerobic Degradation

## Abstract

The *mbd* cluster encodes the anaerobic degradation of 3-methylbenzoate in the β-proteobacterium *Azoarcus* sp. CIB. The specific transcriptional regulation circuit that controls the expression of the *mbd* genes was investigated. The *P_O_*, *P_B_*_1_, and *P*_3_*_R_* promoters responsible for the expression of the *mbd* genes, their cognate MbdR transcriptional repressor, as well as the MbdR operator regions (ATAC*N*_10_GTAT) have been characterized. The three-dimensional structure of MbdR has been solved revealing a conformation similar to that of other TetR family transcriptional regulators. The first intermediate of the catabolic pathway, *i.e.* 3-methylbenzoyl-CoA, was shown to act as the inducer molecule. An additional MbdR-dependent promoter, *P_A_*, which contributes to the expression of the CoA ligase that activates 3-methylbenzoate to 3-methylbenzoyl-CoA, was shown to be necessary for an efficient induction of the *mbd* genes. Our results suggest that the *mbd* cluster recruited a regulatory system based on the MbdR regulator and its target promoters to evolve a distinct central catabolic pathway that is only expressed for the anaerobic degradation of aromatic compounds that generate 3-methylbenzoyl-CoA as the central metabolite. All these results highlight the importance of the regulatory systems in the evolution and adaptation of bacteria to the anaerobic degradation of aromatic compounds.

## Teaser

**Background:** The specific transcriptional regulation of the mbd pathway for anaerobic 3-methylbenzoate degradation is unknown.

**Results:** The MbdR/3-methylbenzoyl-CoA couple controls the induction of the *mbd* genes.

**Conclusion:** MbdR is the regulator of the mbd pathway in *Azoarcus* sp. CIB.

**Significance:** This work highlights the importance of the regulatory systems in the evolution and adaptation of bacteria to the anaerobic degradation of aromatic compounds.

## Introduction

Aromatic compounds are included among the most widespread organic compounds in nature, and some of them are man-made environmental pollutants (1–4). Microorganisms play a fundamental role in the degradation of these aromatic compounds in diverse ecological niches (3, 5–8). Many habitats containing large amounts of aromatic compounds are often anoxic. In the last decades, biochemical studies concerning the anaerobic degradation of aromatic compounds have been steadily accumulating, with benzoyl-CoA representing the intermediate to which most monocyclic aromatic compounds are converted (3–5, 9–12). On the contrary, the study on the specific regulatory systems controlling the expression of the gene clusters involved in the anaerobic degradation of aromatic compounds has been mainly restricted to the characterization of a few transcriptional regulators.

Anaerobic benzoate degradation via benzoyl-CoA was shown to be controlled by the two-component BamVW regulatory system (13) or the BgeR regulator (14) in the obligate anaerobes *Geobacter* strains, and by the BadR/BadM (15, 16) and BzdR/BoxR (17–20) regulators in the facultative anaerobes *Rhodopseudomonas palustris* and *Azoarcus* strains, respectively. Moreover, a few global regulators, *e.g.* AadR, AcpR, and AccR, that influence the anaerobic expression of the benzoyl-CoA central pathway have been reported (15, 21, 22). A TdiSR (TutC1B1) two-component regulatory system was described for the regulation of the *bss/bbs* genes encoding the peripheral pathway that converts toluene into benzoyl-CoA in denitrifying bacteria (4, 23–25). It was also reported that the regulation of the peripheral routes that funnel 4-hydroxybenzoate and *p*-coumarate into the benzoyl-CoA central pathway in the phototrophic *R. palustris* strain is accomplished by the HbaR and CouR proteins, respectively (26, 27). However, no specific-transcriptional regulators that control anaerobic degradation pathways, other than that of benzoyl-CoA and some peripheral routes that converge to the latter, have been described so far.

**FIGURE 1.**
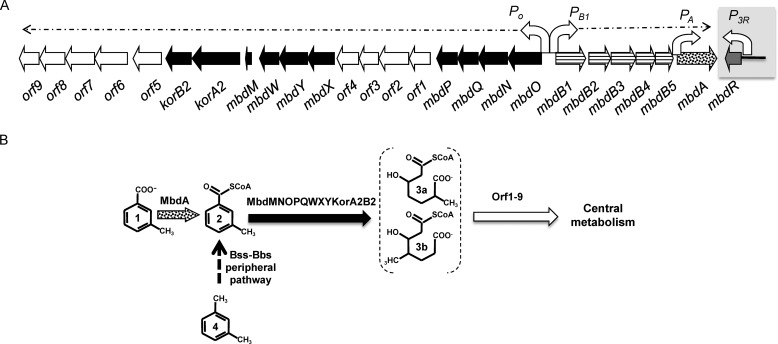
**3-Methylbenzoate anaerobic degradation pathway in *Azoarcus* sp. CIB.** *A*, scheme of the *mbd* gene cluster of *Azoarcus* sp. CIB. Genes are represented by *thick arrows,* and their predicted function is annotated as follows: *gray*, regulatory gene; *horizontal stripes*, genes encoding a 3-methylbenzoate ABC-type transport system; *stippling*, gene encoding the 3-methylbenzoate-CoA ligase; *black*, genes encoding the 3-methylbenzoyl-CoA upper central pathway; *white*, genes involved in the 3-methylbenzoyl-CoA lower pathway (and some genes of unknown function). *Bent arrows* represent the promoters driving the expression of the *mbd* genes. The *mbdO-orf9* operon and the *mbdB1-mbdA* operon are indicated by *broken arrows. B*, scheme of 3-methylbenzoate activation and 3-methylbenzoyl-CoA anaerobic degradation pathway. The enzymes involved are indicated following the same code of *A*. The Bss-Bbs peripheral pathway that converts *m*-xylene into 3-methylbenzoyl-CoA is indicated by a *dashed arrow*. The compounds are as follows: *1*, 3-methylbenzoate; *2*, 3-methylbenzoyl-CoA; *3a*, 3-hydroxy-6-methyl-pimelyl-CoA; *3b*, 3-hydroxy-4-methyl-pimelyl-CoA; and *4*; *m*-xylene.

*Azoarcus* sp. CIB is a denitrifying β-proteobacterium able to anaerobically degrade different aromatic compounds, including some hydrocarbons such as toluene, via benzoyl-CoA, and *m*-xylene, via 3-methylbenzoyl-CoA (28). The *Azoarcu*s sp. CIB *bzd* genes responsible for the anaerobic degradation of benzoate are clustered and consist of the *P_N_* promoter-driven *bzdNOPQMSTUVWXYZA* catabolic operon and the *bzdR* regulatory gene (29). BzdR-mediated repression of *P_N_* is alleviated by the inducer molecule benzoyl-CoA, the first intermediate of the catabolic pathway (17, 18). In addition, the *P_N_* promoter is also subject to control by the benzoyl-CoA-dependent BoxR repressor, a BzdR paralog that regulates the expression of the *box* genes responsible for the aerobic degradation of benzoate in *Azoarcus* sp. CIB (20). The *mbd* cluster of *Azoarcus* sp. CIB encodes the central pathway responsible for the degradation of the 3-methylbenzoyl-CoA formed during the anaerobic degradation of *m*-xylene and 3-methylbenzoate (Fig. 1) (28). The *mbd* cluster is organized in at least three operons, *i.e.* the *mbdO-orf9*, *mbdB1-mbdA,* and *mbdR* operons (Fig. 1*A*). The *mbdB1-mbdA* operon is driven by the *P_B_*_1_ promoter and encodes a putative 3-methylbenzoate ABC transporter (MbdB1B2B3B4B5) and the 3-methylbenzoate-CoA ligase (MbdA) that activates 3-methylbenzoate to 3-methylbenzoyl-CoA (peripheral pathway) (Fig. 1*B*). The *mbdO-orf9* operon is regulated by the *P_O_* promoter and encodes the enzymes for the anaerobic conversion of 3-methylbenzoyl-CoA to a hydroxymethylpimelyl-CoA (MbdMNOPQWXYZ) (upper central pathway) and the further degradation of the latter to the central metabolism (Orf1–9) (lower central pathway) (Fig. 1) (28). The *mbdR* gene was proposed to encode a transcriptional regulator of the TetR family that might regulate the inducible expression of the catabolic *mbd* genes (28). The efficient expression of the *bzd* and *mbd* genes required the oxygen-dependent AcpR activator, and it was under the control of AccR-mediated carbon catabolite repression by some organic acids and amino acids (22, 28).

In this work we have characterized the promoters of the *mbd* cluster and demonstrated the 3-methylbenzoyl-CoA/MbdR-dependent transcriptional control of the *mbd* genes in *Azoarcus* sp. CIB. The studies on the structural-functional relationships of the MbdR protein expand our current view on the transcriptional regulation of anaerobic pathways, and highlight the importance of the regulatory systems in the evolution and adaptation of bacteria to the anaerobic degradation of aromatic compounds.

## EXPERIMENTAL PROCEDURES

### 

#### 

##### Bacterial Strains, Plasmids, and Growth Conditions

Bacterial strains and plasmids used are listed in Table 1. *Escherichia coli* strains were grown in lysogeny broth (LB) medium (31) at 37 °C. When required, *E. coli* cells were grown anaerobically in M63 minimal medium (40) at 30 °C using the corresponding necessary nutritional supplements, 20 mm glycerol, as carbon source, and 10 mm nitrate, as terminal electron acceptor. *Azoarcus* sp. CIB strains were grown anaerobically in MC medium at 30 °C, using the indicated carbon source(s) and 10 mm nitrate as the terminal electron acceptor, as described previously (29). For aerobic cultivation of *Azoarcus* strains, the same MC medium was used but without nitrate. When appropriate, antibiotics were added at the following concentrations: ampicillin (100 μg ml^−1^), gentamicin (7.5 μg ml^−1^), and kanamycin (50 μg ml^−1^).

**TABLE 1:** **Bacterial strains and plasmids used in this study**

Strain or plasmid	Description*^a^*	Ref. or source
***E. coli* strains**		
B834 (DE3)	F^−^, *ompT*, *hsdS_B_*(*r_B_^−^m_B_^−^*), *gal*, *dcm*, *met*, λDE3	30
BL21 (DE3)	F^−^, *ompT*, *hsdS_B_*(*r_B_^−^m_B_^−^*), *gal*, *dcm*, λDE3	31
S17-1λpir	Tp^r^, Sm^r^, *recA*, *thi*, *hsdRM*+, RP4::2-Tc::Mu::Km, Tn*7*, λ*pir* phage lysogen	32
MC4100	*araD139* Δ(*argF-lac*)U169 *rpsL*150 (Sm^r^) *relA1 flbB*5301 *deo*C1 *ptsF25 rbsR*	33

***Azoarcus* sp. strains**		
CIB	Wild-type strain	29
CIBd*mbdR*	Km^r^, CIB mutant strain with a disruption of the *mbdR* gene	This work
CIBd*mbdB1*	Km^r^, CIB mutant strain with a disruption of the *mbdB1* gene	This work
CIBΔ*P_A_*	CIB mutant strain with a deletion of the *P_A_* promoter	This work

**Plasmids**		
pK18*mob*	Km^r^, *ori*ColE1, Mob^+^, *lac*Zα, used for directed insertional disruption	34
pK18mbdRnew	Km^r^, pK18*mob* containing a 524-bp HindIII/EcoRI *mbdR* internal fragment	This work
pK18mbdB1	Km^r^, pK18*mob* containing a 728-bp EcoRI/XbaI *mbdB1* internal fragment	This work
pK18*mobsacB*	Km^r^, *ori*ColE1, Mob^+^, *lac*Zα. Vector with a *sacB* selection marker for gene replacement by double site homologous recombination	34
pK18*mobsacB*Δ*P_A_*	Km^r^, pK18*mobsacB* containing a chimeric 2.6-kb XbaI/HindIII fragment carrying the Δ*P_A_*	This work
pUC19	Ap^R^, *oriColE*1, *lacZ*α	31
pUCmbdA	Ap^R^, pUC19 derivative expressing *mbdA* gene under *Plac* control	28
pIZ1016	Gm^r^, *ori*pBBR1, Mob^+^, *lacZ*α, *Ptac*/*lacI^q^*, broad host range cloning and expression vector	35
pIZP_B1_	Gm^r^, pIZ1016 derivative expressing the *P_B_*_1_::*lacZ* fusion	28
pIZP_A_	Gm^r^, pIZ1016 derivative expressing the *P_A_*::*lacZ* fusion	This work
pIZP_3R_	Gm^r^, pIZ1016 derivative expressing the *P*_3_*_R_*::*lacZ* fusion	This work
pIZmbdA	Gm^r^, pIZ1016 derivative expressing the *mbdA* gene under control of *Ptac*	This work
pCK01	Cm^r^, *ori*pSC101, low copy number cloning vector	36
pCKmbdR	Cm^r^, pCK01 derivative expressing *mbdR* gene under the control of *Plac* promoter	This work
pET-29a(+)	Km^r^, *ori*ColE1, *P_T_*_7_, cloning and overexpression vector	Novagen
pETmbdR	Km^r^, pET-29a (+) expressing *mbdR*-His_6_ under *P_T_*_7_	This work
pEHISTEV	Km^r^, *ori*ColE1, *P_T_*_7_, coding 6His, TEVpro, cloning, and overexpression vector	37
pEHISTEVMbdR	Km^r^, pHISTEV derivative expressing TEV protease-cleavable His_6_ *mbdR* under *P_T_*_7_	This work
pSJ3	Ap^r^, *ori*ColE1, *′lacZ* promoter probe vector	38
pSJ3P_A_	Ap^r^, pSJ3 derivative carrying the *P_A_*::*lacZ* fusion	This work
pSJ3P_3R_	Ap^r^, pSJ3 derivative carrying the *P*_3_*_R_*::*lacZ* fusion	This work
pSJ3P_B1_	Ap^r^, pSJ3 derivative carrying the *P_B_*_1_::*lacZ* fusion	28
pSJ3P_O_	Ap^r^, pSJ3 derivative carrying the *P_O_*::*lacZ* fusion	This work
pJCD01	Ap^r^, *ori*ColE1, polylinker of pUC19 flanked by *rpoC* and *rrnBT*_1_*T2* terminators	39
pJCDP_O_	Ap^r^, pJCD01 derivative harboring a 271-bp ScaI/EcoRI fragment that includes the *P_O_* promoter	This work
pJCDP_B1_	Ap^r^, pJCD01 derivative harboring a 251-bp ScaI/EcoRI fragment that includes the *P_B_*_1_ promoter	This work

*^a^* The abbreviations used are as follows: Ap^r^, ampicillin-resistant; Cm^r^, chloramphenicol-resistant; Gm^r^, gentamicin-resistant; Km^r^, kanamycin-resistant; Sm^r^, streptomycin- resistant; TEV, tobacco etch virus.

##### Molecular Biology Techniques

Standard molecular biology techniques were performed as described previously (31). Plasmid DNA was prepared with a High Pure plasmid isolation kit (Roche Applied Science). DNA fragments were purified with Gene-Clean Turbo (Q-biogene). Oligonucleotides were supplied by Sigma. The oligonucleotides employed for PCR amplification of the cloned fragments and other molecular biology techniques are summarized in Table 2. All cloned inserts and DNA fragments were confirmed by DNA sequencing with fluorescently labeled dideoxynucleotide terminators (41) and AmpliTaq FS DNA polymerase (Applied Biosystems) in an ABI Prism 377 automated DNA sequencer (Applied Biosystems). Transformation of *E. coli* cells was carried out by using the RbCl method or by electroporation (Gene Pulser; Bio-Rad) (31). The proteins were analyzed by SDS-PAGE and Coomassie-stained as described previously (31). The protein concentration was determined by the method of Bradford (42) using bovine serum albumin as the standard. Nucleotide sequence analyses were done at the National Center for Biotechnology Information (NCBI) server (www.ncbi.nlm.nih.gov). Pairwise and multiple protein sequence alignments were made with the ClustalW program (43) at the EMBL-EBI server.

**TABLE 2:** **Oligonucleotides used in this study**

Primers	Sequence (5′ → 3′)*^a^*	Use
CIB+1P_mbdO_3′	CATTTGACGTTCTCCTCCTCACTTG	Primer extension *P_O_* promoter
CIB+1P_mbdB1_3′	CATCTCTCCCTCCTGGACGATGAAG	Primer extension *P_B_*_1_ promoter
PmbdOF1	GCTGGTATGTTGTGCGGAGTGG	Amplification of 203-bp *P_O_* fragment for RT-PCR assays
bcrBR2	TGCGCCATCGTACACTCCTCG	
PmbdB1F1	CGCCGTTTTCCGCAATGACTG	Amplification of 278-bp *P_B_*_1_ fragment for RT-PCR assays
mbdB1R1	GGCAAAGTGGGCGGGCAGC	
PmbdREcoRI 3′	CGGAATTCGTTCCAATGGATTTGCCTCTCGG (EcoRI)	Primer extension *P*_3_*_R_* promoter
PmbdAEcoRI 3′	CGGAATTCCCTCAATGCGCATCAACATAGTG (EcoRI)	Primer extension *P_A_* promoter
5′ mbdRmut2	GCGAAGCTTACCGTGCGACAACGAT (HindIII)	524-bp *mbdR* internal fragment cloned into double-digested pK18*mob* to generate pK18mbdRnew
3′ mbdRmut2	CGGAATTCGCCATTGAGAAGTACCG (EcoRI)	
mbdB1mutEcoRI 5′	GGAATTCCGGCCGCGAGGTTGAGTACG (EcoRI)	728-bp *mbdB1* internal fragment cloned into double-digested pK18*mob* to generate pK18mbdB1
mbdB1mutXbaI 3′	GCTCTAGACCTGCACCGCGTACACGTCG (XbaI)	
P_A_ del. Z1 mbdB4 5′	GCTCTAGACATTTACGGTATTCGAGAACGCG (XbaI)	1191-bp *P mbd_B_* flanking region (Z1) cloned together with Z2 into double-digested pK18*mobsacB* to generate pK18*mobsacB*Δ*P_A_*
P_A_ del. Z1 mbdB5 3′	GGGGTACCTCAAACGCCGAGAAAATTTTTCAAC (KpnI)	
P_A_ del. Z2 Inter. 5′	GGGGTACCGTCACTATGTTGATGCGCATTGAG (KpnI)	1451-bp *P_A_* flanking region (Z2) cloned together with Z1 into double-digested pK18*mobsacB* to generate pK18*mobsacB* Δ*P_A_*
P_A_ del. Z2 mbdA 3′	CCCAAGCTTCAATCTTGAGTACGATCCATGCCTC (HindIII)	
Inter. mbdB5-A 5′	GGGGTACCAAGTTTTCATTATCTCTAGTACCGG (KpnI)	238-bp *mbdB5-mbdA* intergenic fragment including *P_A_* promoter cloned into double-digested pSJ3 to generate pSJ3P_A_
Inter. mbdB5-A 3′.2	GCTCTAGACCCATGGTCGGTTTCCTCAATGCGC (XbaI)	
PmbdRKpnI5′	GGGGTACCATGCTCGAAGTCAGGTATCCATC (KpnI)	451-bp *tdiR-mbdR* intergenic fragment including *P*_3_*_R_* promoter cloned into double-digested pSJ3 to generate pSJ3P_3_*_R_*
PmbdRXbaI3′	GCTCTAGAGGCATGATGTCTGGAGATGTTCC (XbaI)	
PmbdOKpnI5′	GGGGTACCCATCTCTCCCTCCTGGACGATGAAG (KpnI)	563-bp *mbdO-mbdB1* intergenic fragment including *P_O_* promoter cloned into double-digested pSJ3 to generate pSJ3P_O_
PmbdOXbaI3′	GCTCTAGAGGCATTTGACGTTCTCCTCCTCACTTG (XbaI)	
mbdRSalI 5′	ACGCGTCGACTGACCTAAGGAGGTAAATAATGAGAAAGCTGAACAAGAAGGAAG (SalI)	676-bp fragment including *mbdR* gene plus a consensus RBS sequence (double underline) for its cloning into double-digested pCK01 to generate pCKmbdR
mbdRPstI 3′	AACTGCAGTCAGAATGTCGGATTTTTGCAGG (PstI)	
mbdRNdeI 5′	GGAATTCCATATGAGAAAGCTGAACAAGAAGGAAGAGCAGAG (NdeI)	651-bp *mbdR* fragment for its cloning into double-digested pET-29 to generate pETmbdR
mbdRXhoI 3′	CCGCTCGAGGAATGTCGGATTTTTGCAGGAGCC (XhoI)	
mbdRBspHI 5′	GGCGTCATGAGAAAGCTGAACAAGAAG (BspHI)	659-bp *mbdR* fragment for its cloning into double-digested pHISTEV to generate pHISTEVMbdR
mbdRBamHI 3′	ATTCGGATCCTCAGAATGTCGGATTTTTG (BamHI)	
mbdAQ-RT-PCRF3	CCTTAACACCATGCTGACATCG	167-bp *mbdA* fragment amplified in real-time RT-PCR
mbdAQ-RT-PCRR5	CCAGACTTCCGGCAACGTG	
Pdiv>OScaI 5′.2	AAAAGTACTGGTATTACGGTAAGTGCTCCACG (ScaI)	271-bp *mbdO-mbdB1* intergenic fragment including *P_O_* promoter. *P_O_* probe for *in vitro* assays
Pdiv>OEcoRI 3′	CCGGAATTCGCTCCCGCGGCTCTTCCAC (EcoRI)	
Pdiv>B1ScaI 5′.2	AAAAGTACTCGTGGAGCACTTACCGTAATACC (ScaI)	251-bp *mbdO-mbdB1* intergenic fragment including *P_B_*_1_ promoter. *P_B_*_1_ probe for *in vitro* assays
Pdiv>B1EcoRI 3′	CCGGAATTCCCTGCGCGCGGCACTATG (EcoRI)	
PmbdAScaI 5′	AAAAGTACTGAGGCCCCGCCCAAGTTTTC (ScaI)	225-bp *mbdB5-mbdA* intergenic fragment including *P_A_* promoter. *P_A_* probe for *in vitro* assays
PmbdAEcoRI 3′	CGGAATTCCCTCAATGCGCATCAACATAGTG (EcoRI)	
PmbdRScaI 5′	AAAAGTACTCACAACTCTTCACCACCAACGCG (ScaI)	352-bp *tdiR-mbdR* intergenic fragment including *P*_3_*_R_* promoter. *P*_3_*_R_* probe for *in vitro* assays
PmbdREcoRI 3′	CGGAATTCGTTCCAATGGATTTGCCTCTCGG (EcoRI)	
5′POLIIIHK	GGACGCAGTCTTTTGCGTGGTAAC	220-bp internal fragment of housekeeping gene *dnaE* (DNApol III α subunit)
3′POLIIIHK	GTGCGTCAAAGTCGCTGCTGTCG	

*^a^* Engineered restriction sites are underlined, and the corresponding restriction enzyme is shown in parentheses.

##### Synthesis and Purification of 3-Methylbenzoyl-CoA

The 3-methylbenzoyl-CoA was synthesized from the corresponding carboxylic acid via its succinimide ester as described (44). The CoA ester formed was purified by preparative reversed phase HPLC on a 1525 Binary HPLC Pump system (Waters) equipped with a NUCLEOSIL®100–7 C_18_ column (Macherey-Nagel, 50 ml total volume) using acetonitrile in 50 mm potassium phosphate buffer, pH 6.8, at a flow rate of 8 ml min^−1^. The column was equilibrated with 5% acetonitrile; elution was at 25% acetonitrile in buffer. For removal of phosphate, the freeze-dried CoA ester was suspended in 2% aqueous acetonitrile; elution was with 25% aqueous acetonitrile. The purity was checked by reversed phase HPLC as described above and by the UV-visible spectrum. 3-Methylbenzoyl-CoA was stored at −20 °C as freeze-dried powder.

##### Construction of Azoarcus sp. CIBdmbdR and Azoarcus sp. CIBdmbdB1 Mutant Strains

For insertional disruption of *mbdR* and *mbdB1* through single homologous recombination, an internal region of each gene was PCR-amplified with the primer pairs 5′mbdRmut2/3′mbdRmut2 and mbdB1mutEcoRI5′/mbdB1mutXbaI3′ (Table 2). The obtained fragments were double-digested with the appropriate restriction enzymes and cloned into double-digested pK18*mob* vector, generating the pK18mbdRnew and pK18mbdB1 recombinant plasmids (Table 1). These plasmids were transferred from *E. coli* S17-1λ*pir* (donor strain) to *Azoarcus* sp. CIB (recipient strain) by biparental filter mating (32), and exconjugant strains *Azoarcus* sp. CIBd*mbdR* and *Azoarcus* sp. CIBd*mbdB1* were isolated aerobically on kanamycin-containing MC medium harboring 10 mm glutarate as the sole carbon source for counterselection of donor cells. The mutant strains were analyzed by PCR to confirm the disruption of the target genes.

##### Construction of Azoarcus sp. CIBΔP_A_ Mutant Strain

The *P_A_* promoter was deleted by allelic exchange through homologous recombination using the mobilizable plasmid pK18*mobsacB,* which allows positive selections of double-site recombinants using the *sacB* gene of *Bacillus subtilis* (34). In summary, two primer pairs (Table 2) were used to PCR-amplify the 1191-bp (Z1 fragment) and 1451-bp (Z2 fragment) flanking regions of the *P_A_* promoter. Both fragments were digested with restriction endonuclease KpnI and ligated, and the chimeric DNA harboring a deleted *P_A_* promoter was PCR-amplified, double-digested, and cloned into the pK18*mobsacB* plasmid. The resulting pK18*mobsacB*Δ*P_A_* plasmid was transformed into the *E. coli* S17-1λ*pir* strain (donor strain) and then transferred to *Azoarcus* sp. CIB (recipient strain) by biparental filter mating (32). Exconjugants containing first site recombination were selected on kanamycin-containing MC medium harboring 10 mm glutarate as the sole carbon source for counterselection of donor cells. Second site recombination was selected by growth on the same medium supplemented with 5% sucrose and by plating on glutarate-containing MC plates supplemented with 5% sucrose. Correct allelic exchange in sucrose-resistant and kanamycin-sensitive *Azoarcus* sp. CIBΔ*P_A_* was verified by PCR with the appropriate primers (Table 2).

##### Construction of a P_A_::lacZ Translational Fusion

The intergenic region between *mbdB5* and *mbdA* genes that includes the *P_A_* promoter was PCR-amplified using the primers Inter.mbdB5-A5′ and Inter.mbdB5-A3′.2 (Table 2). The resulting 238-bp fragment was KpnI/XbaI double-digested and cloned upstream of the *lacZ* gene into the double-digested pSJ3 promoter probe vector, generating plasmid pSJ3*P_A_* (Table 1). The recombinant pSJ3*P_A_* plasmid was KpnI/HindIII double-digested, and the 3.3-kb fragment containing the *P_A_*::*lacZ* translational fusion was then cloned into the broad host-range pIZ1016 cloning vector (Table 1). To this end, pIZ1016 was KpnI/HindIII double-digested and its *Ptac* promoter and polylinker region were replaced by the *P_A_*::*lacZ* translational fusion, generating plasmid pIZP*_A_* (Table 1).

##### Construction of a P_3R_::lacZ Translational Fusion

The intergenic region between *tdiR* and *mbdR* genes that includes the *P*_3_*_R_* promoter was PCR-amplified using the primers PmbdRKpnI5′ and PmbdRXbaI3′ (Table 2). The resulting 451-bp fragment was KpnI/XbaI double-digested and cloned upstream of a *lacZ* gene into the double-digested pSJ3 promoter probe vector, generating plasmid pSJ3P_3R_ (Table 1). The recombinant pSJ3P_3R_ plasmid was KpnI/HindIII double-digested, and the 3.5-kb fragment containing the *P*_3_*_R_*::*lacZ* translational fusion was then cloned into the broad host range pIZ1016 cloning vector (Table 1). To this end, pIZ1016 was KpnI/HindIII double-digested and its *Ptac* promoter and polylinker region were replaced by the *P*_3_*_R_*::*lacZ* translational fusion, generating plasmid pIZP_3R_ (Table 1).

##### Construction of the pIZmbdA and pCKmbdR Plasmids

The pIZmbdA plasmid is a broad host range plasmid that expresses the *mbdA* gene under the control of the *P_tac_* promoter (Table 1). For the construction of pIZmbdA, the 1.7-kb HindIII/XbaI fragment containing the *mbdA* gene from pUCmbdA (28) was cloned into HindIII/XbaI double-digested pIZ1016 plasmid. The pCKmbdR plasmid (Table 1) expresses the *mbdR* gene under control of the *Plac* promoter in the pCK01 cloning vector. To this end, the *mbdR* gene was PCR-amplified as a 676-bp fragment using mbdRSalI5′ and mbdRPstI3′ oligonucleotides (Table 2). The SalI/PstI double-digested PCR fragment was then cloned into double-digested pCK01 plasmid to generate pCKmbdR.

##### Overproduction and Purification of MbdR

The recombinant pETmbdR plasmid (Table 1) carries the *mbdR* gene, which was PCR-amplified (651-bp) with primers mbdRNdeI5′ and mbdRXhoI3′ (Table 2), with a His_6_ tag coding sequence at its 3′-end, under control of the *P_T_*_7_ promoter that is recognized by the T7 phage RNA polymerase. The gene encoding T7 phage RNA polymerase is present in monocopy in *E. coli* BL21(DE3), and its transcription is controlled by the *Plac* promoter and the LacI repressor, making the system inducible by the addition of isopropyl 1-thio-β-d-galactopyranoside (IPTG).^5^ *E. coli* BL21 (DE3) (pETmbdR) cells were grown at 37 °C in 100 ml of kanamycin-containing LB medium until the culture reached an *A*_600_ of 0.5. Overexpression of the His-tagged protein was then induced during 5 h by the addition of 0.5 mm IPTG. Cells were harvested at 4 °C, resuspended in 10 ml of 20 mm imidazole-containing working buffer (50 mm NaH_2_PO_4_, pH 8, 300 mm KCl), and disrupted by passage through a French press operated at a pressure of 20,000 p.s.i. Cell debris was removed by centrifugation at 16,000 × *g* for 20 min at 4 °C, and the resulting supernatant was used as crude cell extract. The MbdR-His_6_ protein was purified from the crude cell extract by a single-step nickel-chelating chromatography (nickel-nitrilotriacetic acid spin columns, Qiagen). The column was equilibrated with resuspension buffer, loaded with the crude extract, and washed four times with working buffer plus increasing concentrations of imidazole (20, 75, and 100 mm). The MbdR-His_6_ protein was eluted in three steps adding to the column working buffer plus increasing concentrations of imidazole (250 and 500 mm and 1 m). The purity of MbdR-His_6_ protein was analyzed by SDS-12.5% PAGE. When necessary, the protein solutions were dialyzed against working buffer plus 20 mm imidazole, concentrated using Vivaspin 500 columns (Sartorius, 10,000 molecular weight cutoff membrane), and stored at 4 °C where they maintained their activity for at least 6 months.

##### Analytical Ultracentrifugation Methods

Sedimentation velocity and equilibrium were performed to determine the state of association of MbdR-His_6_. The analytical ultracentrifugation analysis was performed using several protein concentrations (from 11 to 46 μm). All samples were equilibrated in buffer containing 50 mm NaH_2_PO_4_, 300 mm KCl, 20 mm imidazole, pH 8. The sedimentation velocity experiments were carried out at 48,000 rpm and 20 °C in an Optima XL-A analytical ultracentrifuge (Beckman-Coulter Inc.) equipped with UV-visible optic detection system, using an An50Ti rotor and 12-mm double sector centerpieces. Sedimentation profiles were registered every 1–5 min at 260 and 275 nm. The sedimentation coefficient distributions were calculated by least squares boundary modeling of sedimentation velocity data using the *c*(*s*) method (45), as implemented in the SEDFIT program. These *s* values were corrected to standard conditions (water at 20 °C and infinite dilution) using the SEDNTERP program (46) to get the corresponding standard *s* values (*s*_20_,*_w_*). Sedimentation equilibrium assays were carried out at speeds ranging from 5000 to 15,000 rpm (depending upon the samples analyzed) and at several wavelengths (260, 280, and 290 nm) with short columns (85–95 μl), using the same experimental conditions and instrument as in the sedimentation velocity experiments. After the equilibrium scans, a high speed centrifugation run (40,000 rpm) was done to estimate the corresponding baseline offsets. The measured low speed equilibrium concentration (signal) gradients of MbdR-His_6_ were fitted using an equation that characterizes the equilibrium gradient of an ideally sedimenting solute (using a MATLAB program, kindly provided by Dr. Allen Minton, National Institutes of Health) to obtain the corresponding buoyant signal average molecular weight.

##### Crystallization and X-ray Crystal Structure Determination of MbdR

To determine the three-dimensional structure of MbdR, the *mbdR* gene from *Azoarcus* sp. CIB was cloned into pEHISTEV vector (37). To this end, the *mbdR* gene was PCR-amplified with primers mbdRBspHI5′ and mbdRBamHI3′ (Table 2) by using genomic DNA of *Azoarcus* sp. CIB as template, digested with BspHI and BamHI, and then ligated into the NcoI/BamHI double-digested pEHISTEV vector, giving rise to plasmid pEHISTEVMbdR. Protein expression of the selenomethionine (SeMet)-substituted recombinant MbdR protein was carried out in *E. coli* B834(DE3) strain (Table 1) transformed with pEHISTEVMbdR, and purification was carried out essentially as described previously (47). The purified SeMet MbdR protein has an extra glycine and alanine at the N terminus resulting from cleavage of the engineered hexa-histidine tag. Crystallization of SeMet MbdR was carried out as described previously (47), and the MbdR crystals were finally grown in the optimized condition of 0.1 m MOPS, pH 7.0, 28% PEG3550, and 0.08% (NH_4_)_2_PO_4_. Structure was determined using SeMet MAD data and refined using CCP4 package (48). The atomic coordinates and structure factors have been deposited in the Protein Data Bank (PDB) under accession number 4uds. Crystallization of MbdR·inducer complex was tried out using the purified MbdR protein with 3-methylbenzoyl-CoA either by co-crystallization or crystal socking, but in both cases the production of crystals failed.

##### RNA Extraction and RT-PCR Assays

*Azoarcus* cells grown in MC medium harboring the appropriate carbon source were harvested at the mid-exponential phase of growth and stored at −80 °C. Pellets were thawed, and cells were lysed in TE buffer (10 Tris-HCl, pH 7.5, 1 mm EDTA) containing 50 mg ml^−1^ lysozyme. Total RNA was extracted using the RNeasy mini kit (Qiagen), including a DNase treatment according to the manufacturer's instructions (Ambion), precipitated with ethanol, washed, and resuspended in RNase-free water. The concentration and purity of the RNA samples were measured by using a, ND1000 spectrophotometer (Nanodrop Technologies) according to the manufacturer's protocols. Synthesis of total cDNA was carried out with 20 μl of reverse transcription reactions containing 400 ng of RNA, 0.5 mm concentrations of each dNTP, 200 units of SuperScript II reverse transcriptase (Invitrogen), and 5 μm concentrations of random hexamers as primers in the buffer recommended by the manufacturer. Samples were initially heated at 65 °C for 5 min then incubated at 42 °C for 2 h, and the reactions were terminated by incubation at 70 °C for 15 min. In standard RT-PCRs, the cDNA was amplified with 1 unit of AmpliTaq DNA polymerase (Biotools) and 0.5 μm concentrations of the corresponding primer pairs (Table 2). Control reactions in which reverse transcriptase was omitted from the reaction mixture ensured that DNA products resulted from the amplification of cDNA rather than from DNA contamination. The *dnaE* gene encoding the α-subunit of DNA polymerase III was used to provide an internal control cDNA that was amplified with oligonucleotides 5′POLIIIHK/3′POLIIIHK (Table 2). The expression of the internal control was shown to be constant across all samples analyzed. For real time RT-PCR assays, the cDNA was purified using the GENECLEAN® Turbo kit (MP Biomedicals), and the concentration was measured using an ND1000 spectrophotometer (Nanodrop Technologies). The IQ5 Multicolor Real Time PCR Detection System (Bio-Rad) was used for real time PCR in a 25-μl reaction containing 10 μl of diluted cDNA (5 ng in each reaction), 0.2 μm primer 5′, 0.2 μm primer 3′, and 12.5 μl of SYBR Green Mix (Applied Biosystems). The oligonucleotides used to amplify a fragment of *mbdA* were mbdAQ-RT-PCRF3 and mbdAQ-RT-PCRR5 (Table 2). PCR amplifications were carried out as follows: 1 initial cycle of denaturation (95 °C for 4 min) followed by 30 cycles of amplification (95 °C, 1 min; test annealing temperature, 60 °C, 1 min; elongation and signal acquisition, 72 °C, 30 s). Each reaction was performed in triplicate. After the PCR, a melting curve was generated to confirm the amplification of a single product. For relative quantification of the fluorescence values, a calibration curve was constructed by 5-fold serial dilutions of an *Azoarcus* sp. CIB genomic DNA sample ranging from 0.5 to 0.5 × 10^−4^ ng. This curve was then used as a reference standard for extrapolating the relative abundance of the cDNA target within the linear range of the curve. Results were normalized relative to those obtained for the *dnaE* internal control.

##### Gel Retardation Assays

DNA probes containing *P_O_*, *P_B_*_1_, *P_A_*, and *P*_3_*_R_* promoters were PCR-amplified with the corresponding primers indicated in Table 2. The amplified DNA was then digested with ScaI and EcoRI restriction enzymes and single end-labeled by filling in the overhanging EcoRI-digested end with [α-^32^]dATP (6000 Ci/mmol; PerkinElmer Life Sciences) and the Klenow fragment of *E. coli* DNA polymerase I as described previously (31). The labeled fragments (*P_O_*, *P_B_*_1_, *P_A_*, and *P*_3_*_R_* probes) were purified using GENECLEAN Turbo (Qbiogen). The retardation reaction mixtures contained 20 mm Tris-HCl, pH 7.5, 10% glycerol, 50 mm KCl, 0.05 nm DNA probe, 250 μg/ml bovine serum albumin, 50 μg/ml unspecific salmon sperm DNA, and purified MbdR-His_6_ protein in a 9-μl final volume. After incubation of the retardation mixtures for 20 min at 30 °C, mixtures were fractionated by electrophoresis in 5% polyacrylamide gels buffered with 0.5× TBE (45 mm Tris borate, 1 mm EDTA). The gels were dried onto Whatman 3MM paper and exposed to Hyperfilm MP (Amersham Biosciences) accompanied by amplifier screens (Cronex Lightning Plus, DuPont). The radioactivity present in the retardation complexes and free probes was quantified by using a densitometer with the Quantity One software (Bio-Rad).

##### DNase I Footprinting Assays

The DNA ^32^P-probes used for these experiments were labeled as indicated for the gel retardation assays. The reaction mixture contained 2 nm DNA probe (*P_O_*, *P_B_*_1_, or *P_A_*), 500 μg/ml bovine serum albumin, and purified MbdR-His_6_ protein in 15 μl of buffer (20 mm Tris-HCl, pH 7.5, 10% glycerol, 50 mm KCl). This mixture was incubated for 20 min at 30 °C, after which 3 μl (0.05 units) of DNase I (Roche Applied Science) (prepared in 10 mm CaCl_2_, 10 mm MgCl_2_, 125 mm KCl, and 10 mm Tris-HCl, pH 7.5) was added, and the incubation was continued at 37 °C for 20 s. The reaction was stopped by the addition of 180 μl of a solution containing 0.4 m sodium acetate, 2.5 mm EDTA, 50 μg/ml salmon sperm DNA, and 0.3 μl/ml glycogen. After phenol extraction, DNA fragments were precipitated with absolute ethanol, washed with 70% ethanol, dried, and directly resuspended in 90% (v/v) formamide-loading gel buffer (10 mm Tris-HCl, pH 8, 20 mm EDTA, pH 8, 0.05% w/v bromphenol blue, 0.05% w/v xylene cyanol). Samples were then denatured at 95 °C for 3 min and fractionated in a 6% polyacrylamide-urea gel. A+G Maxam and Gilbert reactions (49) were carried out with the same fragments and loaded in the gels along with the footprinting samples. The gels were dried onto Whatman 3MM paper and visualized by autoradiography as described previously.

##### Primer Extension Analyses

*Azoarcus* sp. CIB cells were grown anaerobically on MC medium plus 3-methylbenzoate (inducing conditions) or benzoate (control condition) until mid-exponential phase. For the primer extension analysis of *P_O_* and *P_B_*_1_ promoters, total RNA was isolated by using RNeasy mini kit (Qiagen) according to the instructions of the supplier. In the case of *P_A_* and *P*_3_*_R_* promoters, the procedure was the same but *Azoarcus* sp. CIB strains harboring pIZP_A_ or pIZP_3R_ plasmids were used instead of the parental strain due to the weaker nature of these promoters. Primer extension reactions were carried out with the avian myeloblastosis virus reverse transcriptase (Promega) and 15 μg of total RNA as described previously (17), using oligonucleotides CIB+1P_mbdO_3′, CIB+1P_mbdB1_3′, PmbdREcoRI3′, and PmbdAEcoRI3′ (Table 2), which hybridize with the coding strand of the *mbdO*, *mbdB1*, *mbdR,* and *mbdA* genes, respectively. These oligonucleotides were labeled at their 5′-end with phage T4 polynucleotide kinase and [γ-^32^P]ATP (3000 Ci/mmol; PerkinElmer Life Sciences). To determine the length of the primer extension products, sequencing reactions of plasmids pSJ3P_O_, pSJ3P_B1_, pIZP_A_, and pIZP_3R_ (Table 1) were carried out with oligonucleotides CIB+1P_mbdO_3′, CIB+1P_mbdB1_3′, PmbdAEcoRI3′, and PmbdREcoRI3′, respectively, using the T7 sequencing kit and [α^32^P]dATP (PerkinElmer Life Sciences) as indicated by the supplier. Products were analyzed on 6% polyacrylamide-urea gels. The gels were dried on Whatman 3MM paper and exposed to Hyperfilm MP (Amersham Biosciences).

##### In Vitro Transcription Experiments

Multiple-round *in vitro* transcription assays were performed as published previously (50). Plasmids pJCDP_O_ and pJCDP_B1_ (Table 1) were used as supercoiled *P_O_* and *P_B_*_1_ templates. Reactions (50-μl mixtures) were performed in a buffer consisting of 50 mm Tris-HCl, pH 7.5, 50 mm KCl, 10 mm MgCl_2_, 0.1 mm bovine serum albumin, 10 mm dithiothreitol (DTT), and 1 mm EDTA. Each DNA template (0.25 nm) of supercoiled plasmids pJCDP_O_ or pJCDP_B1_ was premixed with 30 nm σ^70^-containing *E. coli* RNA polymerase (1 unit/μl; United States Biochemical Corp.), different amounts of purified MbdR-His_6_ protein, and different concentrations of the 3-methylbenzoyl-CoA inducer. For multiple-round assays, transcription was then initiated by adding a mixture of 500 μm (each) ATP, CTP, and GTP, 50 μm UTP, and 2.5 μCi of [α-^32^P]UTP (3000 Ci/mmol; PerkinElmer Life Sciences). After incubation for 15 min at 37 °C, the reactions were stopped with an equal volume of a solution containing 50 mm EDTA, 350 mm NaCl, and 0.5 mg/ml carrier tRNA. The mRNA produced was then precipitated with ethanol, dissolved in loading buffer (7 m urea, 1 mm EDTA, 0.6 m glycerol, 0.9 mm bromphenol blue, and 1.1 mm xylene cyanol), electrophoresed on a denaturing 7 m urea, 4% polyacrylamide gel, and visualized by autoradiography.

##### β-Galactosidase Assays

The β-galactosidase activities from promoter-*lacZ* reporter fusions were measured with permeabilized cells when cultures reached mid-exponential phase, as described by Miller (40).

## RESULTS

### 

#### 

##### mbdR Gene Encodes a Specific Repressor of the P_O_ and P_B1_ Promoters in Azoarcus sp. CIB

**FIGURE 2.**
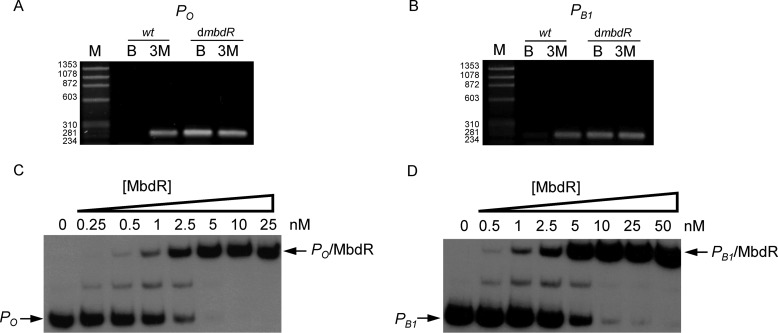
**MbdR protein controls the *P_O_* and *P_B_*_1_ promoters.** *A* and *B*, activity of the *P_O_* and *P_B_*_1_ promoters in wild-type *Azoarcus* sp. CIB and the *Azoarcus* sp. CIBd*mbdR* mutant strain. Agarose gel electrophoresis of RT-PCR products obtained from the divergent promoters *P_O_* (*A*) and *P_B_*_1_ (*B*). Total RNA was extracted from *Azoarcus* sp. CIB (*wt*) and *Azoarcus* sp. CIBd*mbd*R (d*mbd*R) cells grown under denitrifying conditions using 3 mm benzoate (*lane B*) or 3 mm 3-methylbenzoate (*lane 3M*) as sole carbon sources. The primer pairs used to amplify the *mbdO* (*P_O_*) and *mbdB1* (*P_B_*_1_) gene fragments as described under “Experimental Procedures” are detailed in Table 2. *Lane M*, molecular size markers (HaeIII-digested ΦX174 DNA). *Numbers* on the *left* represent the sizes of the markers (in base pairs). *C* and *D*, the MbdR protein binds to the *P_O_* and *P_B_*_1_ promoters. Gel retardation assays were performed as indicated under “Experimental Procedures.” *C* shows the interaction between increasing concentrations of purified MbdR-His_6_ protein and a DNA probe (271-bp) containing the *P_O_* promoter. *D* shows the interaction between increasing concentrations of purified MbdR-His_6_ protein and a DNA probe (251-bp) containing the *P_B_*_1_ promoter. *Lane numbers* refer to the MbdR-His_6_ protein concentration (nanomolar) used for each reaction. *P_O_* and *P_B_*_1_ probes as well as the major *P_O_*·MbdR and *P_B_*_1_·MbdR complexes are marked with *arrows*.

*In silico* analysis at the 3′-end of the *mbd* cluster revealed a gene, *mbdR*, that encodes a putative specific transcriptional regulator (Fig. 1) (28). To analyze the role of the *mbdR* gene in the expression of the catabolic and transport *mbd* genes, an *mbdR* disruptional insertion mutant (*Azoarcus* sp. CIBd*mbdR* strain; Table 1) was constructed. Because *Azoarcus* sp. CIBd*mbdR* mutant strain grew normally on minimal medium containing 3-methylbenzoate as the only carbon source, the *mbdR* gene does not seem to function as a transcriptional activator of the *mbd* genes. Wild-type *Azoarcus* sp. CIB strain and *Azoarcus* sp. CIBd*mbdR* mutant strain were grown anaerobically on minimal medium containing benzoate (control condition) or 3-methylbenzoate (inducing condition) as the only carbon sources, and the expression from *P_O_* and *P_B_*_1_ promoters was analyzed by RT-PCR experiments. Whereas the wild-type strain showed a clear induction of the *P_O_* and *P_B_*_1_ promoters when grown in 3-methylbenzoate, the MbdR mutant exhibited expression from the *P_O_* and *P_B_*_1_ promoters when growing both in benzoate or 3-methylbenzoate (Fig. 2, *A* and *B*). Hence, these results support the idea that MbdR acts as a specific transcriptional repressor of the *P_O_* and *P_B_*_1_ promoters.

##### MbdR Is a New Member of the TetR Family of Transcriptional Regulators

**FIGURE 3.**
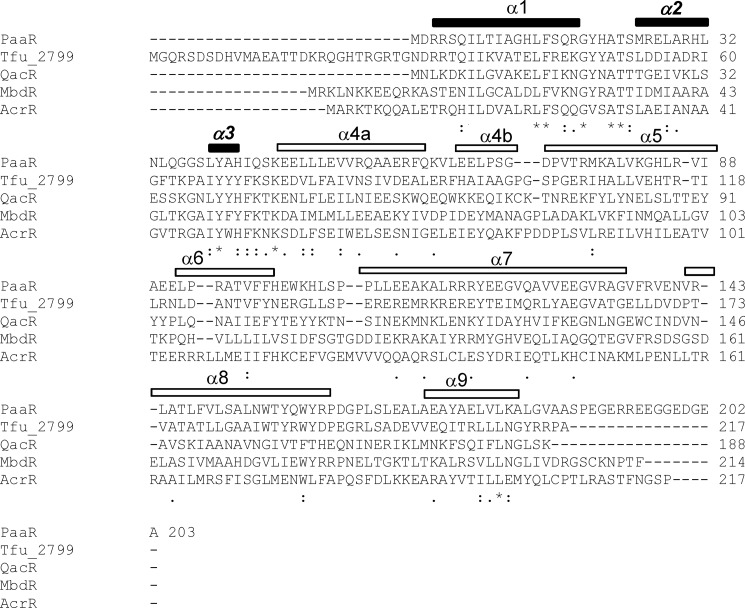
**Multiple sequence alignment of MbdR with other TetR family proteins.** The proteins are as follows: *PaaR*, PaaR regulator from *Thermus thermophilus* HB8 (YP_144239); *Tfu_2799*, TetR-like regulator from *Thermobifida fusca* YX (YP_290855); *QacR*; QacR regulator from *Staphylococcus aureus* (ADK23698); *MbdR*, MbdR regulator from *Azoarcus* sp. CIB (CCH23038); *AcrR*, AcrR regulator from *Salmonella enterica* (AAQ73535). The amino acid residues of each protein are indicated by their standard *one-letter code* and they are *numbered* on the *right*. Sequences were aligned using the multiple sequence alignment program ClustalW. *Asterisks* show identical residues in all sequences. *Dots* indicate conserved residues. The α1–α9 secondary structure elements of the MbdR three-dimensional structure (Protein Data Bank code 4uds) are drawn as *bars* at the *top* of the alignment. The N-terminal α1–α3 helices that constitute the DNA binding domain are shown as *filled bars*, with the helix-turn-helix motif indicated in *bold* and *italics*. The C-terminal α4–α9 helices that constitute the dimerization and ligand binding domain are shown as *open bars*.

Analysis of the primary structure of MbdR shows an overall low amino acid sequence similarity to members of the TetR family of transcriptional regulators (Fig. 3) (51, 52). To determine the structure of the MbdR repressor, we cloned and expressed in the pETmbdR plasmid (Table 1) a C-terminally His-tagged version of the MbdR protein. The MbdR protein (24.9 kDa) was overproduced in *E. coli* BL21 (DE3) cells harboring plasmid pETmbdR and purified from the soluble protein fraction by a single-step affinity chromatography (data not shown). The oligomeric state of MbdR protein in solution was determined by analytical ultracentrifugation experiments carried out at different concentrations (11–46 μm) of MbdR. Sedimentation velocity analysis of 11 μm MbdR revealed a single species with a sedimentation (*s*) value of 2.9 ± 0.1 (data not shown). The molecular mass of the 2.9 S species, as measured by sedimentation equilibrium, is compatible with the mass of the MbdR dimer (data not shown). Because the frictional ratio *f/f*_0_ was 1.46, the shape of the MbdR dimer deviates from that expected for a globular protein and suggests a slightly elongated dimer.

**FIGURE 4.**
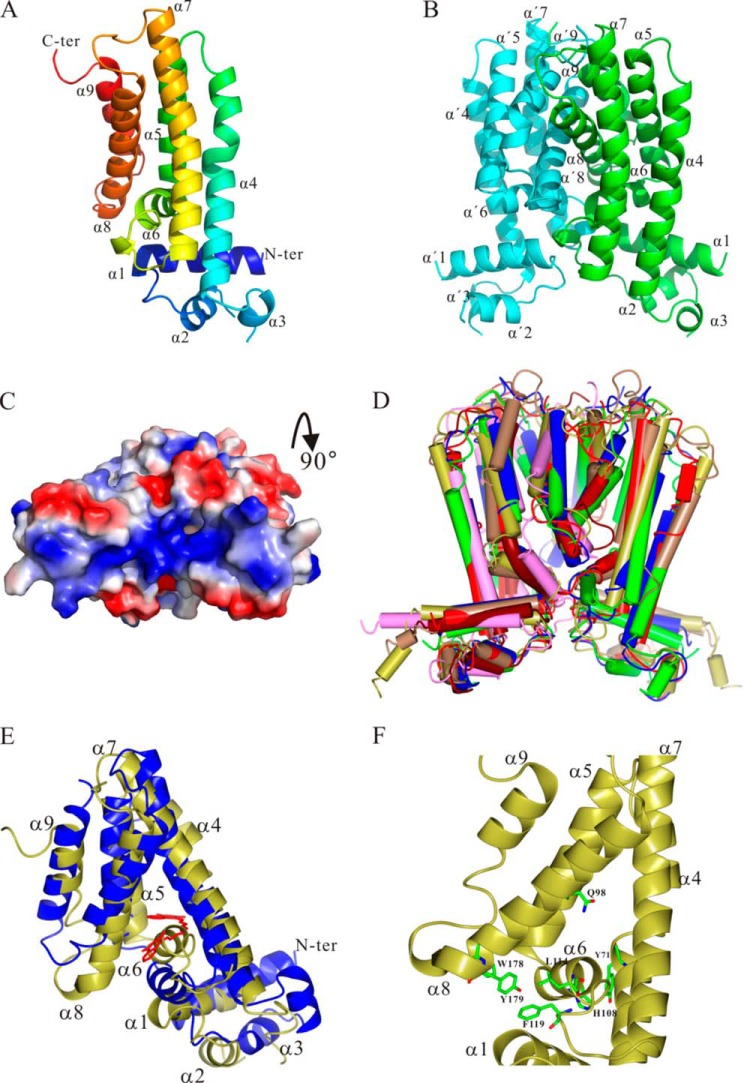
**Three-dimensional structure of MbdR.** *A*, *ribbon* diagram of the three-dimensional structure of the MbdR monomer, which belongs to space group I222. The refined structure has *R*_work_ of 0.185 and *R*_free_ of 0.242 with % completeness of 99.3. *B*, *ribbon* diagram of the MbdR dimer generated using two neighboring monomers, showing the interface and the buried residues. *C*, molecular surface representation of the MbdR dimer with rotation of 90° backward to show the MbdR-DNA interaction surface. *Red* and *blue* surfaces represent negative and positive electrostatic potentials. *D*, similarity (superposition) of MbdR (*red*) to the structures of other TetR-like regulators such as AcrR (3LHQ, *gold*), EthR (3G1O, *tan*), HapR (2PBX, *yellow*), IcaR (2ZCN, *green*), QacR (3BTL, *blue*), and TetR (3LWJ, *cyan*). *E*, superimposition of the MbdR apo-structure (*gold*) and the QacR·4,4′[1,6-hexanediylbis(oxy)]bisbenzenecarboximidamide (*red*) complex structure (*blue*) (3BTJ) to show the proposed internal cavity of MbdR induced by 3-methylbenzoyl-CoA binding. *F,* putative key residues comprising the ligand-binding pocket of MbdR are shown as *sticks.* Figures were drawn using the PyMOL program.

The crystal structure of MbdR was determined using multiple wavelength anomalous diffraction data, and it was refined to 1.76 Å resolution. A summary of the crystallographic statistics is shown in Table 3. The crystal structure reveals that the crystallographic asymmetric unit contains a monomer of the protein (Fig. 4*A*). The N-terminal 14 amino acids, residues Thr-46 and Lys-47, and the C-terminal 10 residues in the structure are disordered. Helices α1 to α3 (Ala-13 to Phe-54) make up the N-terminal DNA binding domain and contain the helix-turn-helix motif (Fig. 3). The larger C-terminal ligand binding domain of MbdR (Fig. 3) consists of helices α4 to α9 (Lys-57 to Val-204) (Fig. 4*A*). The long axis of helices α4, α5, α7, α8, and α9 are approximately parallel and at right angles to α1. The short helix α6 lies approximately parallel to α1 and bisects the C-terminal domain with α4 and α7 on the one side and α5, α8, and α9 on the other side (Fig. 4*A*). A 2-fold crystallographic symmetry operator (arises in space group I222) sits parallel to α4 and generates a dimeric arrangement. The dimer interface is formed mainly by helices α8 and α9 with small contributions from helices α6 and α7. In total, the dimer buries 1759 Å^2^/monomer of surface area with mostly hydrophobic residues (Fig. 4*B*).

**TABLE 3:** **X-ray crystallographic phasing and refinement statistics** Values in parentheses relate to the highest resolution shell.

X-ray source	Diamond Io3
Wavelength (Å)	0.9792
Resolution (Å)	52.35-1.76 (1.8-1.76)
Space group	I222
Unit cell (Å)	*a* = 47.0, *b* = 56.2, *c* = 143.8; α = β = γ = 90
Unique reflections	17,349
Completeness (%)	99.3 (76.7)
Redundancy	7 (6.3)
*R*_merge_ (%)	10.8 (83.5)
*I/*σ (*I*)	17.1 (4.1)
*V_m_* (Å/Da)	1.98 (1mol)
Solvent (%)	37.9

**Refinement**	
*R*_work_/*R*_free_	17.61/22.61 (18.53/24.17)
Figure of merit*^a^*	0.8

**Root mean square deviation**	
Bonds (Å)/angle (°)	0.022/0.74
Average *B*-factor	
All atoms (1677, A^2^)	24.0

**Ramachandran**	
Preferred regions (%)	98.92
Allowed regions (%)	1.08
Outlier (%)	0
PDB code	4uds

*^a^* The figure of merit is calculated after density modification.

Taken together, all these results indicate that the MbdR homodimer shows the characteristic structure of the TetR family regulators. The members of the TetR family are mostly repressors (51, 52), and MbdR behaves also as a transcriptional repressor of the *mbd* genes responsible for the anaerobic catabolism of 3-methylbenzoate.

##### MbdR Binds to Palindrome Operator Sites within P_O_ and P_B1_ Promoters

**FIGURE 5.**
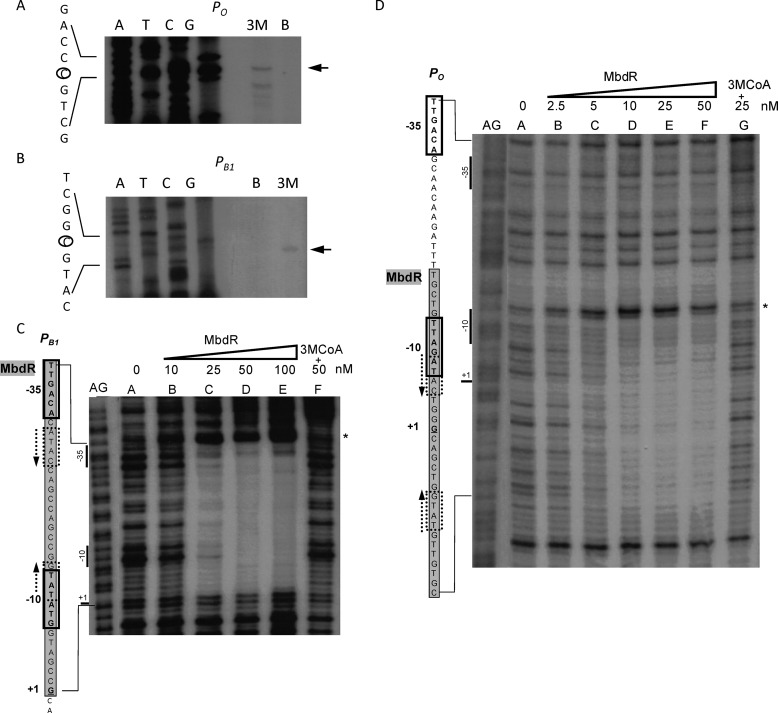
**MbdR protein interacts with the *P_O_* and *P_B_*_1_ promoter regions.** *A* and *B*, determination of the transcription start site in the *P_O_* and *P_B_*_1_ promoters. Total RNA was isolated from *Azoarcus* sp. CIB cells growing on 3-methylbenzoate (inducing condition) or benzoate (control condition) as sole carbon sources as described under “Experimental Procedures.” The size of the extended products under inducing conditions (*lane 3M*) or noninducing conditions (*lane B*) was determined by comparison with the DNA sequencing ladder (*lanes A, T, C,* and *G*) of the *P_O_* (*A*) and *P_B_*_1_ (*B*) promoter regions. Primer extension and sequencing reactions of the *P_O_* and *P_B_*_1_ promoters were performed with primers CIB+1P_mbdO_3′ and CIB+1P_mbdB1_3′, respectively, as described under “Experimental Procedures.” An expanded view of the nucleotides surrounding the transcription initiation site (*circled*) in the noncoding strand is shown. The longest extension product is shown by an *arrow. C* and *D*, DNase I footprinting analyses of the interaction of MbdR with the *P_O_* and *P_B_*_1_ promoter regions. The DNase I footprinting experiments were carried out using the *P_B_*_1_ (*C*) and *P_O_* (*D*) probes labeled as indicated under “Experimental Procedures.” *Lanes AG*, show the A+G Maxam and Gilbert sequencing reaction. *Lanes A–G* show footprinting assays containing increasing concentrations of MbdR-His_6_. *Lanes F* (*C*) and *G* (*D*) show footprinting assays containing MbdR-His_6_ plus 250 μm 3-methylbenzoyl-CoA (*3MCoA*). Phosphodiester bonds hypersensitive to DNase I cleavage are indicated by *asterisks*. On the *left side* of each panel, an expanded view of the promoter region is shown. Protected regions are *shaded* in *gray* over the promoter sequences. The −10/−35 regions are *boxed,* and the transcription initiation sites (+1) are *underlined*. The predicted MbdR operators are flanked by palindrome sequences indicated by convergent *dotted arrows*.

To confirm *in vitro* that the MbdR regulator directly interacts with the *P_O_* and *P_B_*_1_ promoters, gel retardation experiments were carried out with purified MbdR and a 271-bp DNA harboring *P_O_* or a 251-bp DNA containing *P_B_*_1_ as probes. The MbdR protein was able to retard the migration of both DNA probes in a protein concentration-dependent manner (Fig. 2, *C* and *D*). The affinity of MbdR for both *P_O_* and *P_B_*_1_ probes was very similar, showing a relative *K_d_* of 1.71 ± 0.18 and 3.72 ± 0.03 nm, respectively. To further study the interaction of the MbdR protein with the *P_O_* and *P_B_*_1_ promoters, we mapped the transcription start sites of both promoters. Primer extension analyses were performed with total RNA isolated from *Azoarcus* sp. CIB cells grown exponentially in benzoate (control condition) or 3-methylbenzoate (inducing condition). Whereas no transcript was observed from cells growing in benzoate, a transcript band was visible from cells growing in 3-methylbenzoate (Fig. 5, *A* and *B*), confirming a 3-methylbenzoate-dependent activation of the *P_O_* and *P_B_*_1_ promoters. The transcription start site at the *P_O_* and *P_B_*_1_ promoters was mapped at a guanine located 137 and 138 bp upstream of the ATG translation initiation codon of the *mbdO* and *mbdB1* genes, respectively.

To characterize the DNA-binding sites of MbdR within the *P_O_* and *P_B_*_1_ promoters, we performed DNase I footprinting assays. As shown in Fig. 5, *C* and *D*, MbdR protected DNA regions spanning from positions +18 to −16 and from −4 to −34 with respect to the transcription start sites of the *P_O_* and *P_B_*_1_ promoters, respectively. The protected regions contained a conserved palindromic sequence (ATAC*N*_10_GTAT) that is suggested to be the operator sequence recognized by MbdR. The MbdR operator in *P_O_* and *P_B_*_1_ promoters spans the transcription initiation sites as well as the −10 and −35 (only in *P_B_*_1_) sequences for recognition of the σ^70^-dependent RNA polymerase (Fig. 5, *C* and *D*). Therefore, the characterization of the MbdR operator supports the observed repressor role of MbdR at the *P_O_* and *P_B_*_1_ promoters (Fig. 2, *A* and *B*).

##### 3-Methylbenzoyl-CoA Is the Inducer That Alleviates the MbdR-dependent Repression of the mbd Genes

**FIGURE 6.**
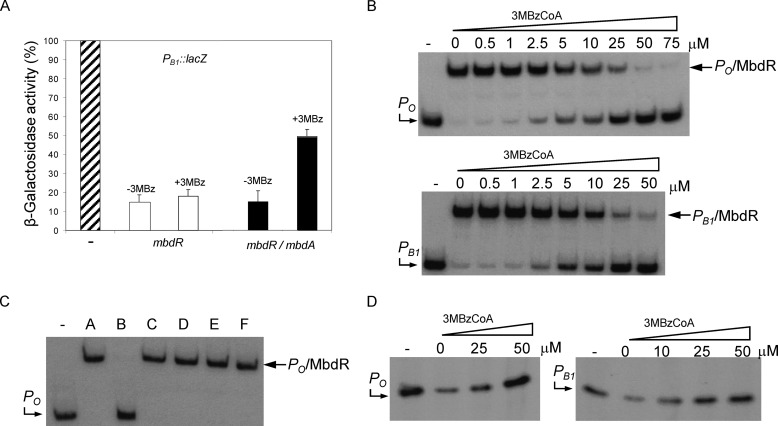
**3-Methylbenzoyl-CoA is the specific inducer of the MbdR regulator.** *A*, expression of the *P_B_*_1_::*lacZ* translational fusion in *E. coli. E. coli* MC4100 cells containing plasmid pIZP_B1_ (*P_B_*_1_::*lacZ*) (*striped bars*), plasmids pIZP_B1_ and pCKmbdR (*mbdR*) (*open bars*), or plasmids pIZP_B1_, pCKmbdR, and pUCmbdA (*mbdA*) (*filled bars*) (Table 1) were grown anaerobically in glycerol-containing minimal medium, supplemented with 0.5 mm IPTG to allow expression of the *mbdR* and *mbdA* genes, in the absence (−3MBz) or presence (+3MBz) of 3 mm 3-methylbenzoate until they reached mid-exponential phase. Values for β-galactosidase activity were determined as indicated under “Experimental Procedures,” and they are represented as a percentage of the activity from *E. coli* MC4100 (pIZP_B1_) cells (4000 Miller units). Each value is the average from three separate experiments (*error bars* indicate S.D.). *B*, interaction of MbdR with the *P_O_* and *P_B_*_1_ promoters in the presence of 3-methylbenzoyl-CoA. Gel retardation assays were performed as indicated under “Experimental Procedures,” and they show the interaction between MbdR-His_6_ protein (30 nm), the *P_O_* (271-bp) or *P_B_*_1_ (251-bp) DNA probes, and increasing concentrations of 3-methylbenzoyl-CoA (*3MBzCoA*). *Lane* −, free *P_O_* and *P_B_*_1_ probes. *Lane numbers* refer to the 3-methylbenzoyl-CoA concentration (μm) used for each assay. *P_O_* and *P_B_*_1_ probes, as well as the *P_O_*·MbdR and *P_B_*_1_·MbdR complexes are marked with *arrows. C,* interaction of MbdR with the *P_O_* promoter in the presence of different aromatic compounds. Gel retardation assays were performed as indicated under “Experimental Procedures,” and they show the interaction between purified MbdR-His_6_ protein (30 nm) and the *P_O_* probe in the absence (*lane A*) or presence (*lanes B–F*) of different aromatic compounds: *lane B*, 250 μm 3-methylbenzoyl-CoA; *lane C*, 2 mm benzoyl-CoA; *lane D*, 2 mm phenylacetyl-CoA; *lane E*, 2 mm 3-methylbenzoate; *lane F*, 2 mm 3-methylbenzoate + 2 mm CoA. *Lane* −, free *P_O_* probe. The *P_O_* probe and the *P_O_*·MbdR complex are marked with *arrows. D*, effect of MbdR and 3-methylbenzoyl-CoA on *in vitro* transcription from *P_O_* and *P_B_*_1_. Multiple-round *in vitro* transcription reactions were performed as indicated under “Experimental Procedures” by using pJCDP_O_ and pJCDP_B1_ plasmid templates (Table 1) that produce 227- and 224-nucleotide mRNAs (*arrows*) from *P_O_* and *P_B_*_1_ promoters, respectively, and 30 nm *E. coli* RNA polymerase. The transcription reactions were carried in the absence of repressor (*lanes* −) or in the presence of 100 nm MbdR-His_6_ with increasing concentrations of 3-methylbenzoyl-CoA (*3MBzCoA*). *Lane numbers* refer to the 3-methylbenzoyl-CoA concentration (μm) used for each assay.

To identify the inducer molecule that alleviates the specific repression exerted by MbdR on the expression of the *mbd* genes, we first accomplished an *in vivo* approach. Thus, the activity of a *P_B_*_1_::*lacZ* translational fusion in plasmid pIZP_B1_ (Table 1) was measured in *E. coli* cells harboring also the pCKmbdR plasmid that expresses the *mbdR* gene under the IPTG-controlled *Plac* promoter (Table 1). As shown in Fig. 6*A*, the β-galactosidase activity levels of recombinant *E. coli* cells expressing the *mbdR* gene and grown anaerobically in minimal medium with glycerol as sole carbon source were significantly lower than those obtained in *E. coli* control cells lacking the *mbdR* gene. This result confirms in a heterologous host the role of MbdR as a transcriptional repressor of the *mbd* genes. Interestingly, the addition of 3-methylbenzoate to the culture medium of recombinant *E. coli* cells unable to metabolize this aromatic acid did not alleviate the repression exerted by MbdR (Fig. 6*A*), suggesting that 3-methylbenzoate, the substrate of the mbd pathway, is not the specific inducer of the *P_B_*_1_ promoter. It has been described previously that the transcriptional activation of benzoate degradation operons in *Azoarcus* sp. CIB requires benzoyl-CoA, the first intermediate of the anaerobic/aerobic degradation pathways, as inducer molecule (17, 20). Thus, we checked whether 3-methylbenzoyl-CoA, the first CoA-derived intermediate of the mbd pathway, could be the specific inducer molecule of the *mbd* genes. To this end, we expressed the *mbdA* gene encoding the 3-methylbenzoate-CoA ligase (MbdA) that catalyzes the transformation of 3-methylbenzoate to 3-methylbenzoyl-CoA (28), in the reporter *E. coli* strain containing plasmids pIZP_B1_ and pCKmbdR. As shown in Fig. 6*A*, the activity of the *P_B_*_1_ promoter increased after the addition of 3-methylbenzoate to the culture medium, suggesting that 3-methylbenzoyl-CoA is the specific inducer of the MbdR repressor.

*In vitro* experiments were then performed to confirm the direct role of 3-methylbenzoyl-CoA as the inducer molecule of the *mbd* cluster. First, gel retardation experiments showed that the presence of 3-methylbenzoyl-CoA inhibited the interaction of MbdR with the *P_O_* and *P_B_*_1_ probes (Fig. 6*B*). On the contrary, 3-methylbenzoate or some 3-methylbenzoyl-CoA analogs, such as benzoyl-CoA or phenylacetyl-CoA, did not avoid the interaction of MbdR with its target promoters (Fig. 6*C*), suggesting that MbdR recognizes 3-methylbenzoyl-CoA specifically. The inducing effect of 3-methylbenzoyl-CoA was also observed in footprinting assays where the addition of 3-methylbenzoyl-CoA reverted the protection of MbdR against the DNase I digestion on the *P_O_* and *P_B_*_1_ promoters (Fig. 5, *C* and *D*).

The role of MbdR as a specific transcriptional repressor of the *P_O_* and *P_B_*_1_ promoters and 3-methylbenzoyl-CoA as the cognate inducer was also demonstrated by *in vitro* transcription assays using supercoiled DNA templates bearing each of the two promoters. Thus, Fig. 6*D* shows the MbdR-dependent repression of the *P_O_* and *P_B_*_1_ promoters, and it also reveals how the addition of increasing amounts of 3-methylbenzoyl-CoA leads to formation of the expected transcripts from both promoters.

##### Identification of Additional MbdR-dependent Promoters in the mbd Cluster, the P_3R_ and P_A_ Promoters

**FIGURE 7.**
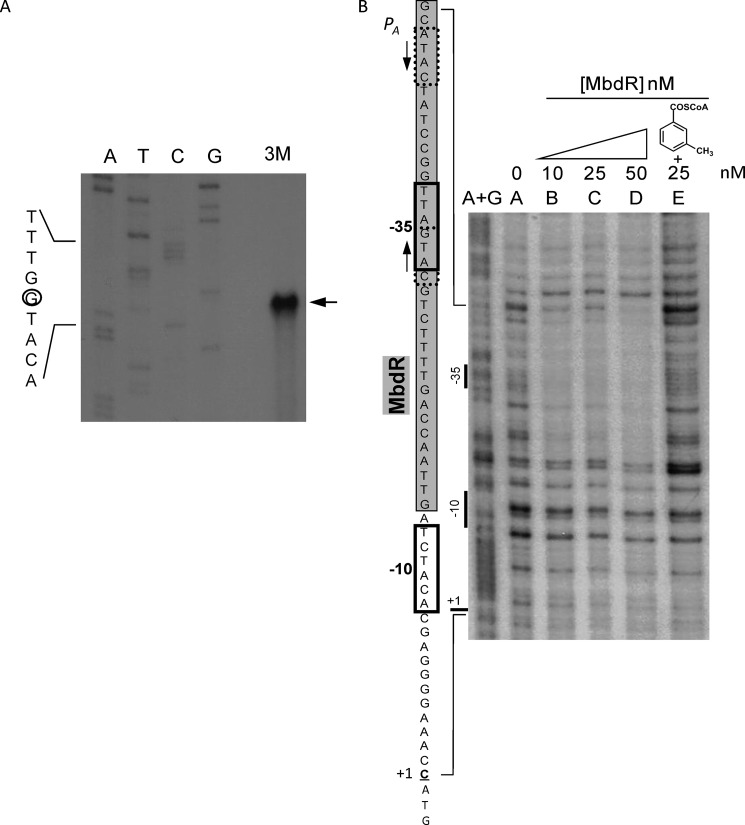
**MbdR protein interacts with the *P_A_* promoter region.** *A*, determination of the transcription start site at the *P_A_* promoter. Total RNA was isolated from *Azoarcus* sp. CIB cells growing on 3-methylbenzoate (*lane 3M*) as sole carbon source as described under “Experimental Procedures.” The size of the extended product was determined by comparison with the DNA sequencing ladder (*lanes A, T, C,* and *G*) of the *P_A_* promoter region. Primer extension and sequencing reactions of the *P_A_* promoter were performed with primer PmbdAEcoRI3′ (Table 2), as described under “Experimental Procedures.” An expanded view of the nucleotides surrounding the transcription initiation site (*circled*) in the noncoding strand is shown. The longest extension product is pointed by an *arrow. B*, DNase I footprinting analyses of the interaction of purified MbdR protein and the *P_A_* promoter region. The DNase I footprinting experiments were carried out using the *P_A_* probe labeled as indicated under “Experimental Procedures.” *Lane A*+*G* shows the A+G Maxam and Gilbert sequencing reaction. *Lanes A–D* show footprinting assays containing increasing concentrations of MbdR-His_6_. *Lane E* shows a footprinting assay containing MbdR-His_6_ (25 nm) in the presence of 250 μm 3-methylbenzoyl-CoA. *Left side*, an expanded view of the *P_A_* promoter region is shown. The protected region is *shaded in gray* over the promoter sequence. The −10/−35 regions are *boxed,* and the transcription initiation site (+1) is *underlined*. The predicted MbdR operator is flanked by palindrome sequences indicated by *convergent arrows*.

Nucleotide sequence analysis of the intergenic regions of the *mbd* cluster revealed putative MbdR binding regions that contain the conserved (ATAC*N*_10_GTAT) palindromic sequence in the *P*_3_*_R_* promoter that drives the expression of *mbdR* (Fig. 1) (28) and upstream of the *mbdA* gene encoding the 3-methylbenzoate-CoA ligase (putative *P_A_* promoter). To experimentally validate that *P*_3_*_R_* and *P_A_* are functional promoters of the *mbd* cluster, the upstream region of *mbdR* and the *mbdB5-mbdA* intergenic region were cloned into the promoter probe vector pSJ3, rendering plasmids pSJ3P_3R_ and pSJ3P_A_ that contain the *P*_3_*_R_*::*lacZ* and *P_A_*::*lacZ* translational fusions, respectively (Table 1). Both translational fusions were then subcloned into the broad host range vector pIZ1016 giving rise to plasmids pIZP_3R_ (*P*_3_*_R_*::*lacZ*) and pIZP_A_ (*P_A_*::*lacZ*) (Table 1). *E. coli* cells containing plasmids pIZP_3R_ or pIZP_A_ were grown in M63 minimal medium, and they showed 75 and 50 Miller units of β-galactosidase activity, respectively, suggesting that *P*_3_*_R_* and *P_A_* are functional but weak promoters. Primer extension experiments revealed that the transcription initiation sites (+1) of *P*_3_*_R_* and *P_A_* promoters are located 120 bp (data not shown) and 117 bp (Fig. 7*A*) upstream of the *mbdR* and *mbdA* start codons, respectively.

**FIGURE 8.**
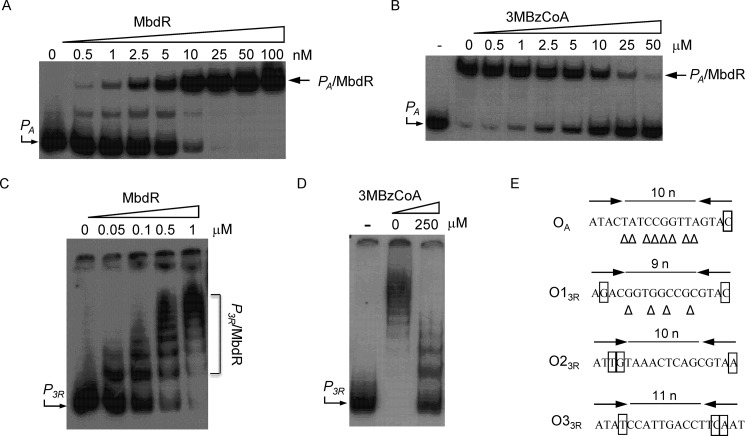
**MbdR protein binds to the *P_A_* and *P*_3_*_R_* promoters and 3-methylbenzoyl-CoA acts as inducer.** Gel retardation assays were performed as indicated under “Experimental Procedures.” *A* shows the interaction between increasing concentrations of purified MbdR-His_6_ protein and a DNA probe (225-bp) containing the *P_A_* promoter. *B* shows the interaction between MbdR-His_6_ protein (30 nm), the *P_A_* DNA probe, and increasing concentrations of 3-methylbenzoyl-CoA (*3MBzCoA*). *Lane* −, free *P_A_* probe. *Lanes 0* to *100* (*A*) and *0* to *50* (*B*) refer to the MbdR-His_6_ protein concentration (nm) and the 3-methylbenzoyl-CoA concentration (μm) used for each assay, respectively. *P_A_* probe as well as the major *P_A_*·MbdR complex are marked with *arrows. C* shows the interaction between increasing concentrations of purified MbdR-His_6_ protein and a DNA probe (352-bp) containing the *P*_3_*_R_* promoter. *Lanes 0* to *1* refer to the MbdR-His_6_ protein concentration (μm) used for each reaction. *P*_3_*_R_* probe as well as the *P*_3_*_R_*·MbdR complexes are marked with an *arrow* and a *bracket*, respectively. *D* shows the interaction between MbdR-His_6_ protein (0.5 μm), the *P*_3_*_R_* DNA probe, and 0 or 250 μm of 3-methylbenzoyl-CoA (*3MBzCoA*). *Lane* −, free *P*_3_*_R_* DNA probe. *E*, nucleotide sequence of the predicted MbdR operator regions in promoters *P_A_* (*O_A_*) and *P*_3_*_R_* (*O1*_3_*_R_*, *O2*_3_*_R_*, and *O3*_3_*_R_*). The flanking ATAC and GTAT palindrome regions are indicated by *convergent arrows*, and the nonconserved nucleotides are *boxed*. Nucleotides that extend the palindromic regions are indicated by *triangles*.

To demonstrate the direct interaction of MbdR with the *P*_3_*_R_* and *P_A_* promoters, gel retardation assays were performed. To this end, purified MbdR was incubated either with a 352-bp DNA probe carrying the *P*_3_*_R_* promoter or with a 225-bp DNA fragment containing the *P_A_* promoter. Fig. 8, *A* and *C*, shows that MbdR was able to retard the migration of both DNA probes in a protein concentration-dependent manner. The binding was specific, because the addition of unlabeled heterologous DNA did not affect the protein-DNA binding, but the addition of unlabeled specific DNA inhibited the retardation of the probes (data not shown). Several *P*_3_*_R_*-MbdR retardation bands were observed (Fig. 8*C*), which agrees with the fact that several MbdR operator regions were suggested in *P*_3_*_R_* (Fig. 8*E*). As observed previously with the *P_O_* and *P_B_*_1_ promoters, 3-methylbenzoyl-CoA behaved as the inducer of MbdR because binding of this protein to the *P_A_* and *P*_3_*_R_* promoters was significantly diminished in the presence of this aromatic CoA ester (Fig. 8, *B* and *D*).

Although the role of *P*_3_*_R_* driving the expression of the *mbdR* regulatory gene is obvious, the role of the *P_A_* promoter located within the *P_B_*_1_-driven operon (Fig. 1) is puzzling, and therefore, it was further investigated.

##### P_A_ and P_B1_ Promoters Are Essential for Growth of Azoarcus sp. CIB on 3-Methylbenzoate

As described previously, the *P_B_*_1_ promoter drives the expression of the *mbdB1B2B3B4B5mbdA* operon (Fig. 1) (28). We have shown above (Fig. 8*A*) that a new MbdR-dependent promoter, the *P_A_* promoter, is located upstream of *mbdA* within the *P_B_*_1_-driven operon (Fig. 1). To explore whether both promoters share a similar MbdR-dependent regulation, the sequence of the *P_A_* promoter recognized by MbdR was experimentally determined by DNase I footprinting assays. Fig. 7*B* shows that the region of *P_A_* protected by MbdR against the DNase I digestion includes the predicted (ATAC*N*_10_GTAC) operator region (Fig. 8*E*), and it spans the −35 sequence for recognition of the σ^70^-dependent RNA polymerase. Moreover, the addition of 3-methylbenzoyl-CoA released the MbdR-dependent protection (Fig. 7*B*), confirming the role of this molecule as inducer. All these data support the hypothesis that MbdR behaves also as a transcriptional repressor for the *P_A_* promoter. To confirm *in vivo* the repressor role of MbdR on the *P_A_* promoter, the activity of a *P_A_*::*lacZ* translational fusion in plasmid pIZP_A_ (Table 1) was measured in *E. coli* MC4100 cells harboring also the pCKmbdR and pUCmbdA plasmids that express the *mbdR* and *mbdA* genes under the IPTG-controlled *Plac* promoter, respectively (Table 1). The β-galactosidase activity levels (5 Miller units) of recombinant *E. coli* cells expressing the *mbdR/mbdA* genes and grown anaerobically were significantly lower than those obtained in *E. coli* control cells expressing the *P_A_*::*lacZ* translational fusion but lacking the *mbdR/mbdA* genes (50 Miller units). However, the addition of 3-methylbenzoate to the culture medium, which is transformed to 3-methylbenzoyl-CoA by the MbdA activity, alleviated the repression exerted by MbdR, and values of β-galactosidase activity of about 40 Miller units were obtained. Therefore, these results show that MbdR behaves as a functional repressor of the *P_A_* promoter, and 3-methylbenzoyl-CoA acts as the inducer molecule.

**FIGURE 9.**
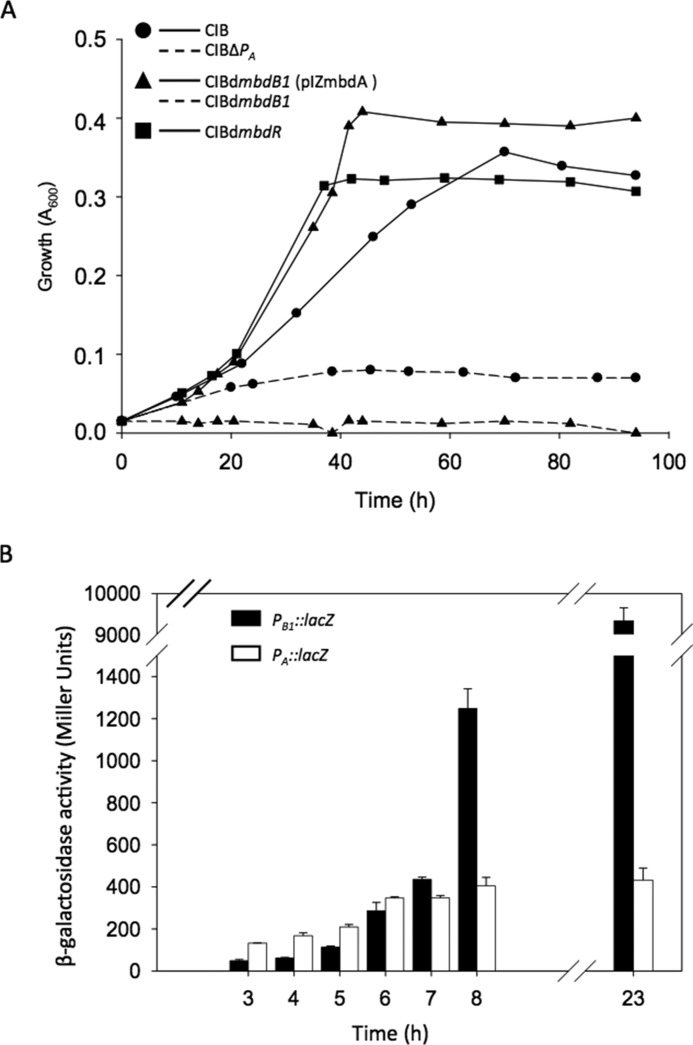
**Role of the *P_B_*_1_ and *P_A_* promoters of the *mbd* cluster in *Azoarcus* sp. CIB.** *A*, growth curves of *Azoarcus* sp. CIB (*solid line*, *circles*), *Azoarcus* sp. CIBΔ*P_A_* (*dotted line, circles*), *Azoarcus* sp. CIBd*mbdB1* (*dotted line, triangles*), *Azoarcus* sp. CIBd*mbdB1* (pIZmbdA) (*solid line*, *triangles*), and *Azoarcus* sp. CIBd*mbdR* (*solid line*, *squares*) (Table 1) growing anaerobically in MC medium containing 3 mm 3-methylbenzoate as detailed under “Experimental Procedures.” *B*, β-galactosidase activity of *Azoarcus* sp. CIB cells harboring plasmids pIZP_B1_ (*P_B_*_1_::*lacZ*) (*closed bars*) or pIZP_A_(*P_A_*::*lacZ*) (*open bars*). Cells were grown anaerobically in MC minimal medium with 7 mm glutarate until the cultures reached an *A*_600_ ∼0.6, and then diluted to *A*_600_ ∼0.17 and induced with 3 mm 3-methylbenzoate. β-Galactosidase activity (in Miller units) was measured at different time points (h) after induction as described under “Experimental Procedures.” *Error bars* represent standard deviation of at least three independent experiments.

As suggested above by comparing the β-galactosidase values in *E. coli* cells expressing *P_A_*::*lacZ* (50 Miller units) and *P_B_*_1_::*lacZ* (4000 Miller units) fusions, the *P_A_* promoter appears to be significantly weaker than *P_B_*_1_. To confirm the major role of *P_B_*_1_ in the expression of the *mbdA* gene in the homologous system, we checked by real time RT-PCR the expression of *mbdA* in the wild-type *Azoarcus* sp. CIB strain and in *Azoarcus* sp. CIBd*mbdB1*, a mutant strain that contains an insertion within the *mbdB1* gene that should block transcription from the *P_B_*_1_ promoter but maintains a functional *P_A_* promoter (Table 1). The expression levels of the *mbdA* gene in *Azoarcus* sp. CIBd*mbdB1* grown in the presence of 3-methylbenzoate were similar to the basal levels observed with the wild-type CIB strain grown in the absence of 3-methylbenzoate, and they were more than 47 times lower than those observed in the wild-type CIB strain grown in 3-methylbenzoate (data not shown). These data suggested that *P_B_*_1_, but not *P_A_*, has indeed a major contribution to the *mbdA* expression in *Azoarcus* sp. CIB. In agreement with this observation, the *Azoarcus* sp. CIBd*mbdB1* mutant strain was unable to use 3-methylbenzoate as sole carbon source (Fig. 9*A*), and growth was restored when the *mbdA* gene was provided in *trans* in plasmid pIZmbdA (Fig. 9*A*). In contrast, *Azoarcus* sp. CIBd*mbdB1* mutant strain was still able to use *m*-xylene as a sole carbon source (data not shown), which is in agreement with the fact that the Bss-Bbs peripheral pathway for the anaerobic degradation of *m*-xylene generates 3-methylbenzoyl-CoA without the need of a specific CoA ligase activity (Fig. 1) (53–56). Taken together, all of these results indicated that *P_B_*_1_ is essential for growth of *Azoarcus* sp. CIB in 3-methylbenzoate by providing an efficient expression of the *mbdA* gene rather than by transcribing the *mbdB1-B5* genes encoding a putative 3-methylbenzoate ABC transporter.

Nevertheless, the presence of the *P_A_* promoter within the *P_B_*_1_-driven operon raised a question about the role of this weak promoter in 3-methylbenzoate degradation. To confirm whether *P_A_* is essential for the anaerobic degradation of 3-methylbenzoate, an *Azoarcus* sp. CIBΔ*P_A_* mutant strain harboring a deletion of the *P_A_* promoter but maintaining a complete *mbdA* gene and the native *P_B_*_1_ promoter was constructed (Table 1). Interestingly, *Azoarcus* sp. CIBΔ*P_A_* was not able to grow anaerobically in 3-methylbenzoate (Fig. 9*A*), suggesting that *P_A_* is also necessary for an efficient expression of the *mbdA* gene, which in turn supports the presence of *P_A_* within the *P_B_*_1_-driven operon.

Because *P_B_*_1_ accounts for most of the *mbdA* expression, the role of the weak *P_A_* promoter might be related to the initial induction of the *mbdA* expression when the cells start to grow in 3-methylbenzoate. To check this hypothesis, the activity of the *P_B_*_1_ and *P_A_* promoters was analyzed by β-galactosidase assays along the growth curve of *Azoarcus* sp. CIB harboring pIZ*P_B_*_1_ (*P_B_*_1_::*lacZ*) and *Azoarcus* sp. CIB harboring pIZP_A_ (*P_A_*::*lacZ*) grown in the presence of 3-methylbenzoate. The activity of the weak *P_A_* promoter was always higher than that of *P_B_*_1_ up to 6 h after the addition of 3-methylbenzoate, and then *P_B_*_1_ showed a significant induction and reached values about 20-fold higher than those of *P_A_* (Fig. 9*B*). Therefore, these results suggest that the fast and modest induction of the *P_A_* promoter will be critical to provide the required amount of the inducer molecule 3-methylbenzoyl-CoA for triggering the induction of the *P_B_*_1_ promoter and to allow growth on 3-methylbenzoate.

## DISCUSSION

Bacterial metabolism of some compounds that usually are nonpreferred carbon sources, *e.g.* aromatic compounds, is generally strictly regulated at the transcriptional level (8). In this work, we have characterized the specific regulation of the *mbd* central cluster, which is responsible for anaerobic 3-methylbenzoate degradation in *Azoarcus* sp. CIB, by the MbdR transcriptional repressor. MbdR is an efficient repressor of the *mbd* genes whose expression can only be switched on when the *Azoarcus* sp. CIB cells grow anaerobically on 3-methylbenzoate (28) but not on benzoate (Fig. 2, *A* and *B*). This finding provides an explanation to the fact that *Azoarcus* sp. CIBd*bzdN*, a strain lacking a functional benzoate degradation (*bzd*) pathway, cannot use benzoate anaerobically despite the Mbd enzymes that can activate benzoate to benzoyl-CoA and further metabolize this CoA-derived compound (28). On the other hand, it is worth noting that the *bzd* genes are not induced when *Azoarcus* sp. CIB grows anaerobically in 3-methylbenzoate (data not shown). Therefore, these results reveal that there is no cross-induction between the bzd and mbd pathways, supporting the existence of devoted BzdR- and MbdR-dependent regulatory systems that control, respectively, each of these two central catabolic pathways in *Azoarcus* sp. CIB.

Analytical ultracentrifugation and crystallographic data indicate that MbdR is a homodimer in solution, a common feature shared by most TetR-like regulators (Fig. 4*D*) (51, 52). Like other members of the TetR family, *e.g.* TetR (57), QacR (58), ActR (59), FadR (60, 61), PfmR (62), and the MbdR monomeric structure includes two domains with nine helices (α1 to α9) linked by loops (Fig. 4*A*). The N-terminal DNA binding domain (helices α1 to α3) contains the helix-turn-helix motif whose amino acid sequence is rather conserved in other TetR-like transcriptional regulators (Fig. 3). An electrostatic surface representation of the MbdR dimer structure shows a positively charged patch at the N-terminal domain of both monomers (Fig. 4*C*), which might contact the phosphate backbone of the target operator region as in the cases of other TetR family proteins (52). An 18-bp conserved palindromic sequence (ATAC*N*_10_GTAT) was suggested to be the operator region recognized by MbdR in *P_O_* and *P_B_*_1_ promoters (Fig. 5). The MbdR binding regions in *P_O_* and *P_B_*_1_ promoters span the transcription initiation sites as well as the −10 and −35 (only in *P_B_*_1_) sequences for recognition of the σ^70^-dependent RNA polymerase (Fig. 5, *C* and *D*), which is in agreement with the observed repressor role of MbdR at both promoters (Fig. 2, *A* and *B*), and it supports MbdR as a transcriptional repressor of the *mbd* cluster. Although the length of the MbdR operator region is similar to that of other TetR operators, their different consensus sequences agree with the fact that the DNA-binding mechanisms differ among the TetR family proteins (52).

*In vivo* (Fig. 6*A*) and *in vitro* (Fig. 6, *B* and *D*) experiments revealed that 3-methylbenzoyl-CoA, the first intermediate of the mbd catabolic pathway, is the cognate inducer molecule that interacts with the MbdR repressor allowing transcription from the *P_O_* and *P_B_*_1_ promoters. There is an increasing number of regulators, *i.e.* PaaR (63) (TetR family), CouR, FerC, HcaR, FerR, and GenR (MarR family) (27, 64–67), PaaX (GntR family) (38), and BzdR and BoxR(XRE family) (17, 20), that control aromatic degradation pathways and recognize aromatic CoA thioesters as inducers. Thus, FerR/FerC recognize feruloyl-CoA; CouR/HcaR recognize *p*-coumaroyl-CoA; BzdR/BoxR/GenR recognize benzoyl-CoA; and PaaX/PaaR recognize phenylacetyl-CoA. In this work, we show that MbdR constitutes the first member of this group of regulators that belongs to the TetR family and controls the anaerobic catabolism of aromatic compounds.

The C-terminal domain of TetR-like regulators is highly variable, with its specific surfaces required for the dimerization of the protein and for the interaction with the inducer (51, 52, 57). Based on the previously published studies of other TetR-like regulators, ligand binding usually induces a conformational change in the protein that leads to changes in DNA recognition and interaction, causing the dissociation of the repressor from the cognate promoter (52). To date, all ligands bind in the same general location at or near the dimer interface. However, it has been shown that in some members of the TetR family, for example AcrR (68), the ligand binds in a large internal cavity in the C-terminal region, surrounded by helices α4 through α8 of each monomer. In contrast, MbdR and other members of TetR family, such as QacR (58), do not have such a cavity (Fig. 4, *A* and *C*). By superimposing the apo-MbdR structure with the structure of the QacR·diamidine hexamidine complex (69), we could suggest the binding site of 3-methylbenzoyl-CoA in MbdR and a model of the MbdR-3-methylbenzoyl-CoA interaction (Fig. 4*E*). Binding of 3-methylbenzoyl-CoA would require the movements of helices α5, α6, α8, and α9 in MbdR, similar to that described as the “induced fit” mechanism of QacR bound to its ligand (69, 70). Similar to what has been observed in the QacR·ligand complex structure, the movement of α6 after 3-methylbenzoyl-CoA binding to MbdR would induce a rotation of the helix-turn-helix domain (Fig. 4*E*), and as a consequence, this DNA binding domain would lose its DNA binding ability. Sequence comparison of MbdR and PaaR (Fig. 3), another member of the TetR family which uses phenylacetyl-CoA as inducer (63), shows two MbdR-specific hydrophobic clusters, Gln-107 to Gly-123 within α6 and the α6/α7 linkage loop, and Ser-165 to Ile-176 within α8. Some residues within these two clusters could be involved in discriminating between the 3-methylbenzoyl group of 3-methylbenzoyl-CoA and the phenylacetyl group of phenylacetyl-CoA (Fig. 4*F*). Nevertheless, further experiments are needed to determine the structure of the MbdR·3-methylbenzoyl-CoA complex for understanding the inducer specificity determinants and the molecular mechanism of transcriptional de-repression at the target promoters.

*P_A_* and *P*_3_*_R_* are two additional promoters within the *mbd* cluster whose activity levels are lower than those of *P_O_* and *P_B_*_1_ but that share with the latter the 3-methylbenzoyl-CoA/MbdR-dependent control (Fig. 8). The *P*_3_*_R_* promoter drives the expression of the regulatory *mbdR* gene (Fig. 1). Interestingly, the amount of MbdR needed for the retardation of 50% of the *P*_3_*_R_* probe was at least 1 order of magnitude higher than that needed for the retardation of the *P_A_* (Fig. 8*A*), *P_O_* (Fig. 2*C*), and *P_B_*_1_ (Fig. 2*D*) promoters. The fact that the activity from the *P*_3_*_R_* promoter is under auto-repression by MbdR at high protein concentrations underlines the importance of a negative feedback loop that would restrict the intracellular concentration of the transcriptional repressor when it reaches a given concentration. The *P_A_* promoter is located within the *P_B_*_1_-driven operon (Fig. 1). The predicted MbdR operator region (ATAC*N*_10_GTAT) (Fig. 8*E*) spans the −35 sequence for recognition of the σ^70^-dependent RNA polymerase in the *P_A_* promoter (Fig. 7*B*), thus supporting the observed repressor role of MbdR on this promoter. Whereas the role of *P*_3_*_R_* driving the expression of the *mbdR* regulatory gene is obvious, the role of the *P_A_* promoter was puzzling, and therefore, it was further investigated.

Inactivation of either the strong (*P_B_*_1_) or the weak (*P_A_*) promoters in *Azoarcus* sp. CIBd*mbdB1* and *Azoarcus* sp. CIBΔ*P_A_* mutant strains, respectively, revealed that both promoters are essential for the anaerobic growth of strain CIB in 3-methylbenzoate (Fig. 9*A*). However, whereas *P_B_*_1_ accounts for most of the *mbdA* expression when the cells are actively growing in 3-methylbenzoate, the *P_A_* promoter allows the initial induction of the *mbdA* expression when the cells start to grow in this aromatic compound (Fig. 9*B*). Therefore, these results suggest that the fast and modest induction of the *P_A_* promoter in the presence of 3-methylbenzoate leads to an increase of *mbdA* expression that, in turn, would enhance the amount of the inducer molecule 3-methylbenzoyl-CoA triggering the induction of the *P_B_*_1_ promoter. The expression of the *mbdA* gene driven by the induced *P_B_*_1_ promoter will provide the required amount of MbdA for the efficient degradation of 3-methylbenzoate and thus will allow growth on this aromatic compound. In summary, these studies highlight the main role of some minor regulatory loops that control the expression of CoA ligases for triggering the efficient expression of aromatic catabolic pathways that use aryl-CoA compounds as central intermediates.

Mbd enzymes are able to activate benzoate and further convert benzoyl-CoA *in vitro* (28). We have shown here that MbdR is an efficient repressor of the *mbd* genes, and it recognizes 3-methylbenzoyl-CoA, but not benzoyl-CoA, as inducer. These results suggest that the broad substrate range *mbd* catabolic genes have recruited a regulatory system based on the MbdR regulator and its target promoters to evolve to a distinct central aromatic catabolic pathway that is only expressed for the anaerobic degradation of aromatic compounds that generate 3-methylbenzoyl-CoA as central metabolite. Thus, the existence in *Azoarcus* sp. CIB of two different central pathways, *i.e.* the *bzd* pathway, for the anaerobic degradation of aromatic compounds that generate benzoyl-CoA as central intermediate, and the *mbd* pathway, for the anaerobic degradation of aromatic compounds that generate 3-methylbenzoyl-CoA as central intermediate, could be mainly driven by the high specificity of the corresponding repressors, *i.e.* BzdR and MbdR, for their cognate inducers, *i.e.* benzoyl-CoA and 3-methylbenzoyl-CoA, respectively. If correct, this highlights the importance of the regulatory systems in the evolution and adaptation of bacteria to the anaerobic degradation of aromatic compounds.

The studies presented in this work expand our knowledge on the specific regulation of anaerobic pathways for the catabolism of aromatic compounds (4, 9, 14, 17, 20, 27, 28). Moreover, it worth noting that 3-methylbenzoyl-CoA is an uncommon metabolite in living cells, and MbdR-responsive promoters are likely to be also very infrequent in nature. Therefore, the *P_B_*_1_ promoter, *mbdR* regulator, and *mbdA* genes become potential BioBricks for creating new conditional expression systems that respond to 3-methylbenzoate in a fashion minimally influenced by the host and that has no impact on the host physiology (biological orthogonality), two major desirable traits in current synthetic biology approaches (71).

## Footnotes

5 The abbreviations used are:

IPTG: isopropyl 1-thio-β-d-galactopyranoside
SeMet: selenomethionine.
